# Predictive Big Data Analytics: A Study of Parkinson’s Disease Using Large, Complex, Heterogeneous, Incongruent, Multi-Source and Incomplete Observations

**DOI:** 10.1371/journal.pone.0157077

**Published:** 2016-08-05

**Authors:** Ivo D. Dinov, Ben Heavner, Ming Tang, Gustavo Glusman, Kyle Chard, Mike Darcy, Ravi Madduri, Judy Pa, Cathie Spino, Carl Kesselman, Ian Foster, Eric W. Deutsch, Nathan D. Price, John D. Van Horn, Joseph Ames, Kristi Clark, Leroy Hood, Benjamin M. Hampstead, William Dauer, Arthur W. Toga

**Affiliations:** 1 Statistics Online Computational Resource, School of Nursing, Michigan Institute for Data Science, University of Michigan, Ann Arbor, Michigan, United States of America; 2 Institute for Systems Biology, Seattle, Washington, United States of America; 3 Information Sciences Institute, University of Southern California, Los Angeles, California, United States of America; 4 Computation Institute, University of Chicago and Argonne National Laboratory, Chicago, Illinois, United States of America; 5 Stevens Neuroimaging and Informatics Institute, University of Southern California, Los Angeles, California, United States of America; 6 Department of Psychiatry and Michigan Alzheimer’s Disease Center, University of Michigan, Ann Arbor, Michigan, United States of America; 7 Veterans Affairs Ann Arbor Healthcare System, Ann Arbor, Michigan, United States of America; 8 Udall Center of Excellence for Parkinson’s Disease Research, University of Michigan, Ann Arbor, Michigan, United States of America; Centre Hospitalier Universitaire Vaudois, SWITZERLAND

## Abstract

**Background:**

A unique archive of Big Data on Parkinson’s Disease is collected, managed and disseminated by the Parkinson’s Progression Markers Initiative (PPMI). The integration of such complex and heterogeneous Big Data from multiple sources offers unparalleled opportunities to study the early stages of prevalent neurodegenerative processes, track their progression and quickly identify the efficacies of alternative treatments. Many previous human and animal studies have examined the relationship of Parkinson’s disease (PD) risk to trauma, genetics, environment, co-morbidities, or life style. The defining characteristics of Big Data–large size, incongruency, incompleteness, complexity, multiplicity of scales, and heterogeneity of information-generating sources–all pose challenges to the classical techniques for data management, processing, visualization and interpretation. We propose, implement, test and validate complementary model-based and model-free approaches for PD classification and prediction. To explore PD risk using Big Data methodology, we jointly processed complex PPMI imaging, genetics, clinical and demographic data.

**Methods and Findings:**

Collective representation of the multi-source data facilitates the aggregation and harmonization of complex data elements. This enables joint modeling of the complete data, leading to the development of Big Data analytics, predictive synthesis, and statistical validation. Using heterogeneous PPMI data, we developed a comprehensive protocol for end-to-end data characterization, manipulation, processing, cleaning, analysis and validation. Specifically, we (i) introduce methods for rebalancing imbalanced cohorts, (ii) utilize a wide spectrum of classification methods to generate consistent and powerful phenotypic predictions, and (iii) generate reproducible machine-learning based classification that enables the reporting of model parameters and diagnostic forecasting based on new data. We evaluated several complementary model-based predictive approaches, which failed to generate accurate and reliable diagnostic predictions. However, the results of several machine-learning based classification methods indicated significant power to predict Parkinson’s disease in the PPMI subjects (consistent accuracy, sensitivity, and specificity exceeding 96%, confirmed using statistical n-fold cross-validation). Clinical (e.g., Unified Parkinson's Disease Rating Scale (UPDRS) scores), demographic (e.g., age), genetics (e.g., rs34637584, chr12), and derived neuroimaging biomarker (e.g., cerebellum shape index) data all contributed to the predictive analytics and diagnostic forecasting.

**Conclusions:**

Model-free Big Data machine learning-based classification methods (e.g., adaptive boosting, support vector machines) can outperform model-based techniques in terms of predictive precision and reliability (e.g., forecasting patient diagnosis). We observed that statistical rebalancing of cohort sizes yields better discrimination of group differences, specifically for predictive analytics based on heterogeneous and incomplete PPMI data. UPDRS scores play a critical role in predicting diagnosis, which is expected based on the clinical definition of Parkinson’s disease. Even without longitudinal UPDRS data, however, the accuracy of model-free machine learning based classification is over 80%. The methods, software and protocols developed here are openly shared and can be employed to study other neurodegenerative disorders (e.g., Alzheimer’s, Huntington’s, amyotrophic lateral sclerosis), as well as for other predictive Big Data analytics applications.

## Introduction

### Big Data challenges, and predictive analytics

There is no unifying theory, single method, or unique set of tools for Big Data science. This is due to the volume, complexity, and heterogeneity of such datasets, as well as fundamental gaps in our knowledge of high-dimensional processes where distance measures degenerate (curse of dimensionality) [1, 2]. To solidify the theoretical foundation of Big Data Science, significant progress is required to further develop core principles of distribution-free and model-agnostic methods to achieve accurate scientific insights based on Big Data datasets. IBM’s 4V’s of “Big Data” (volume, variety, velocity and veracity) provide a qualitative descriptive definition of such datasets. We use an alternative approach to constructively define “Big Data” and explicitly describe the challenges, algorithms, processes, and tools necessary to manage, aggregate, harmonize, process, and interpret such data. The six defining characteristics of Big Data are large size, incongruency, incompleteness, complexity (e.g., data format), multiplicity of scales (from micro to meso to macro levels, across time, space and frequency spectra), and multiplicity of sources. Predictive Big Data analytics refers to algorithms, systems, and tools that use Big Data to extract information, generate maps, prognosticate trends, and identify patterns in a variety of past, present or future settings. The core barriers to effective, efficient and reliable predictive Big Data analytics are directly related to these six distinct Big Data attributes and highlight two critical challenges. The first is that Big Data increases faster (Kryder’s law) than our ability to computationally handle it (Moore’s law) [3]. Storage capacity doubles every 1.2–1.4 years [4], whereas the number of transistors per fixed volume doubles every 1.5–1.7 years [5].The second is that the energy (value) of fixed Big Data decreases exponentially from the point of its acquisition. This leads to substantial loss of resources (e.g., reduced data life-span, “data exhaust”) and enormous missed opportunities (e.g., lower chances of alternative efforts) [6, 7].

### Neurodegenerative disorders

Age-related central nervous system (CNS) neurodegenerative disease is a rapidly growing societal and financial burden [8]. Alzheimer’s disease (AD), Parkinson’s disease (PD) and amyotrophic lateral sclerosis (ALS), which together affect over six million Americans [9–11], are some the three most serious illnesses in this category. The incidence of these debilitating diseases increases sharply with advancing age which, together with the rapid aging of developed societies, is creating a crisis of human suffering and enormous economic pressure. Abnormal deposition of protein, which manifests as intra- or extracellular protein aggregates, is a unifying feature of these disorders. Although distinct proteins aggregate in each illness, they assume a common abnormal structure known as amyloid. Amyloid formation is a stepwise process whereby small numbers of misfolded proteins form “seeds,” which greatly accelerate kinetics of subsequent amyloid formation. It is increasingly recognized that these misfolded proteins also have the capacity to spread from cell to cell in a “prion” like manner [12]. Thus, amyloid formation within cells may lead to synaptic dysfunction due to deposition of insoluble peptides and bioactive oligomers [13], and this protein-based insult may be linked to disease progression through intra-cellular spreading. As more biospecimen data, including pathological reports [14] and amyloid-based positron emission tomography (PET) imaging [15], are collected and preprocessed, they can easily be fused and harmonized with the complex data we use in this study to provide additional biomelecular evidence for the detection, tracking and prognosis of neurodegeneration using Big Data analytics.

### Recent PD studies

**Fig 1:**
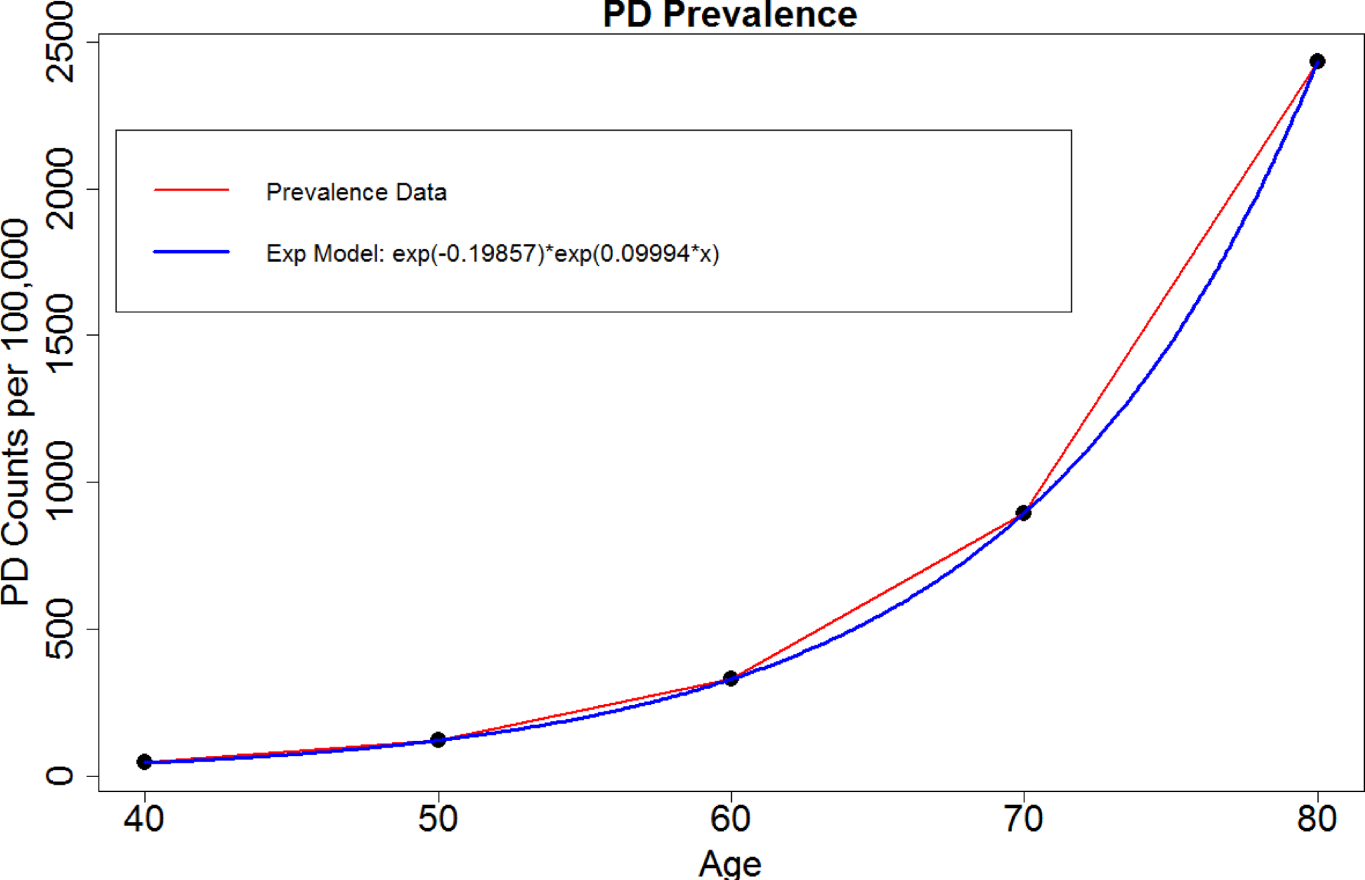
Exponential growth model of PD prevalence with age.

A number of recent studies have examined the relation of Parkinson’s disease to trauma [16], genetics [17], environment [18] and other co-morbidities [19]. A recent meta-analytic study [20] pooled published data from 1985 to 2010 and analyzed the effects of age, geographic location, and gender on PD prevalence. The authors identified substantial difference between cohorts using 47 manuscripts and showed a rising prevalence of PD with age (per 100,000), **Fig 1**. Significant differences in prevalence by sex were found in 50 to 59 year-olds, with a prevalence of 41 in females and 134 in males. Previously, we have developed and validated automated pipeline workflows to extract imaging biomarkers and explore associations of imaging and phenotypic data in Parkinson’s and Alzheimer’s disease [3, 21]. A study of normocholinergic and hypocholinergic PD patients examined the basal forebrain-cortical and pedunculopontine nucleus-thalamic cholinergic projection systems and reported that a combination of rapid eye movement sleep behavior disorder (RBD) symptoms and fall history yielded diagnostic accuracy of 81% for predicting combined thalamic and cortical cholinergic deficits [22]. Discoveries of familial PD kinase leucine-rich repeat kinase 2 (LRRK2) protein, α-Synuclein locus duplication [13], and mouse models of Parkinson’s provide clues for understanding the impact of various signaling events on neurodegeneration [23–25].

### Study goals and innovation

This study utilizes PPMI neuroimaging, genetics, clinical and demographic data to develop classification and predictive models for Parkinson’s disease. Specifically, we aim to aggregate and harmonize all the data, jointly model the entire data, test model-based and model-free predictive analytics, and statistically validate the results using n-fold cross validation. Some of the challenges involved in such holistic predictive Big Data analytics include the sampling incongruency (e.g., non-corresponding spatio-temporal observations) and heterogeneity (e.g., types, formats) of the data elements, incompleteness of the data, complexities regarding the representation of complementary components in the data (e.g., image intensities are in 3D Cartesian coordinates, whereas clinical, genetic and phenotypic data elements are represented in alternative bases). We tested generalized linear models (with fixed and random effects) as well as multiple classification methods to discriminate between Parkinson’s disease patients and asymptomatic healthy controls (HC). Previous studies have reported results of integrating multiple types of data to diagnose, track and predict Parkinson’s disease using imaging and genetics [26, 27], genome-wide association studies [28], animal phenotypic models [29], molecular imaging [30], pharmacogenetics [31], phenomics and genomics [32]. However, few studies have reported strategies to efficiently and effectively handle all available multi-source data to produce high-fidelity predictive models of this neurodegenerative disorder, which is the focus of this investigation. The main contributions of this study include (i) an approach for rebalancing initially imbalanced cohorts, (ii) applying a wide spectrum of automated classification methods that generate consistent and powerful phenotypic predictions (e.g., diagnosis), (iii) developing a reproducible machine-learning based protocol for classification that enables the reporting of model parameters and outcome forecasting, and (iv) using generalized estimating equations models to assess population-wide differences based on incomplete longitudinal Big Data. Reproducible protocols that enable such end-to-end data analytics are critical to inform future scientific investigations, enable discovery-based Big Data science, facilitate active trans-disciplinary collaborations, and entice independent community validation of algorithmic modules, atomic tools, and complete end-to-end workflows.

## Methods

### Study Design and Approach

**Fig 2:**
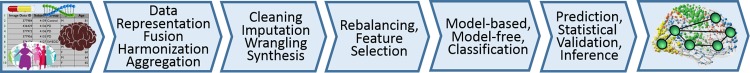
Overview of the complete analytics protocol (from data handling, to pre-processing, modeling, classification and forecasting).

As we deal with large, complex, multi-source, incomplete and heterogeneous data, to obtain valid and robust diagnostic forecasting predictions, our approach needs to start with efficient data handling, manipulation, processing, aggregation, and harmonization. This includes methods for identification of missing patterns, data wrangling, imputation, conversion, fusion and cross-linking. Next, we need to introduce mechanisms for automated extraction of structured data elements representing biomedical signature vectors associated with unstructured data (e.g., images, sequences). This includes processing the sequences to extract specific genotypes and deriving neuroimaging signature vectors as proxies of global and regional brain organization. The subsequent data modeling and diagnostic prediction demands a high-throughput and flexible interface to model-based and model-free techniques that can be applied to the harmonized and aggregated multivariate data. We have employed the statistical computing environment R for our model fitting, parameter estimation and machine learning classification. The final component of this protocol requires a computational platform that enables each of these steps to be implemented, integrated and validated. To ensure the success of the entire process, enable internal validation, and assure external reproducibility of the results, the Pipeline environment was chosen to support the critical element of integrating, productizing, demonstrating, and testing the entire processing protocol as a pipeline workflow. **Fig 2**illustrates a top-level schematic of our end-to-end protocol. The following sub-sections describe the data characteristics, the specific manipulation, processing, cleaning, analysis, and protocol validation steps involved in this study.

### The PPMI initiative

Between 2002 and 2015, The Michael J. Fox Foundation for Parkinson’s Research (MJFF) has funded about $90 million in biomarker research. MJFF-PPMI (Parkinson’s Progression Markers Initiative) collaboration involves researchers, industry, government and study participants that conducts an observational clinical study to verify progression markers in Parkinson’s disease. PPMI efforts aim to establish a comprehensive set of clinical, imaging and biosample markers that may be used to diagnose, track and predict PD and its progression. To accelerate biomarker research and validation, PPMI acquires, aggregates and shares a large and comprehensive set of clinical data and biospecimens that are made available to the entire scientific community. PPMI data and specimens are collected using standardized protocols developed by the Initiative’s steering committee. The PPMI study dataset includes clinical, biological and imaging data collected at multiple participating sites. The data are assembled into the PPMI study database and distributed by the PPMI Bioinformatics Core at the University of Southern California. PPMI also collects biologic specimens including urine, plasma, serum, cerebrospinal fluid, DNA and RNA. These are available for research purposes via study biorepositories in the US, Italy and Israel (http://www.ppmi-info.org/access-data-specimens).

### Data

The complete PPMI data archive includes demographics (e.g., age, medical history), clinical tests (physical, verbal learning and language, neurological and olfactory (University of Pennsylvania Smell Identification Test, UPSIT) tests), vital signs, MDS-UPDRS scores (Movement Disorder Society–Unified Parkinson's Disease Rating Scale), ADL (activities of daily living), Montreal Cognitive Assessment (MoCA), Epworth Sleepiness Scale, REM sleep behavior questionnaire, Geriatric Depression Scale (GDS-15), and State-Trait Anxiety Inventory for Adults. In this study, we used imaging, demographic, genetic and clinical data from the PPMI repository (http://www.ppmi-info.org/access-data-specimens).

**Fig 3:**
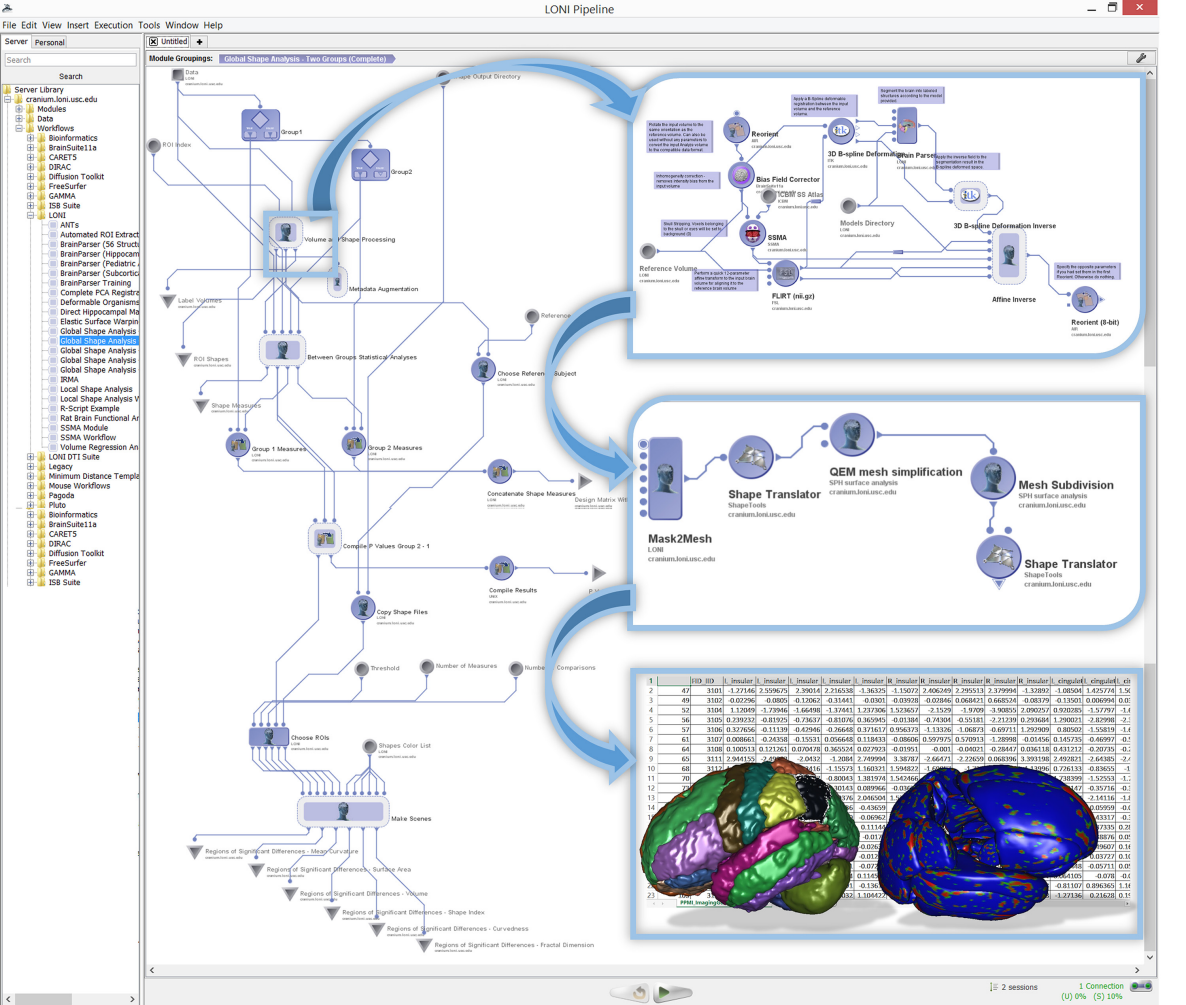
Overview of the global shape analysis (GSA) pipeline workflow for automated extraction of 280 neuroimaging biomarkers for 28 regions within each brain hemisphere and five complementary shape morphometry metrics. Insert images illustrate examples of the nested processing steps and the textual, visual and statistical output generated by the pipeline protocol.

The baseline structural magnetic resonance imaging (sMRI) data included 3D T1-weighted sequences (MPRAGE or SPGR) with total scan time between 20–30 min and covered the vertex, cerebellum and pons. The T1-weighted sagittal images had slice thickness of less than 1.5mm and no interslice gaps. Voxel dimensions were 1 × 1 × 1.2*mm*^3^ with a voxel matrix of size 256 × 256 × *Z*, where 170 ≤ *Z* ≤ 200. Derived imaging data (imaging biomarkers) were obtained by pre-processing and analyzing the sMRI data using the global shape analysis (GSA) pipeline workflow [33, 34]. The GSA protocol uses demographics and imaging data to implement a study design involving group-based extraction, modeling and analysis of regional brain anatomy. This pipeline workflow (http://bit.ly/1DjhkG9) computes 6 global shape measures (mean-curvature, surface area, volume, shape-index, curvedness and fractal dimension) for each of 28 automatically extracted regions of interest (ROIs) within both brain hemispheres. Between-cohort statistics using shape morphometry measures are calculated and the results include 3D scenes showing ROI models of the cohort statistical differences. **Fig 3**shows the main components of the GSA pipeline workflow.

The PPMI genetics data includes over 600 samples that passed initial DNA quality control (QC), over 300 samples run on the Illumina ImmunoChip (interrogating 196,524 variants) and NeuroX (covering 240,000 exonic variants, of which 24,000 specific to neurodegeneration) with 100% sample success rate, and 98.7% genotype success rate [35]. These samples were genotyped for APOE e2/e3/e4 (derived from single nucleotide polymorphism (SNPs) rs7412 and rs429358) using TaqMan genotyping [36].

**Fig 4:**
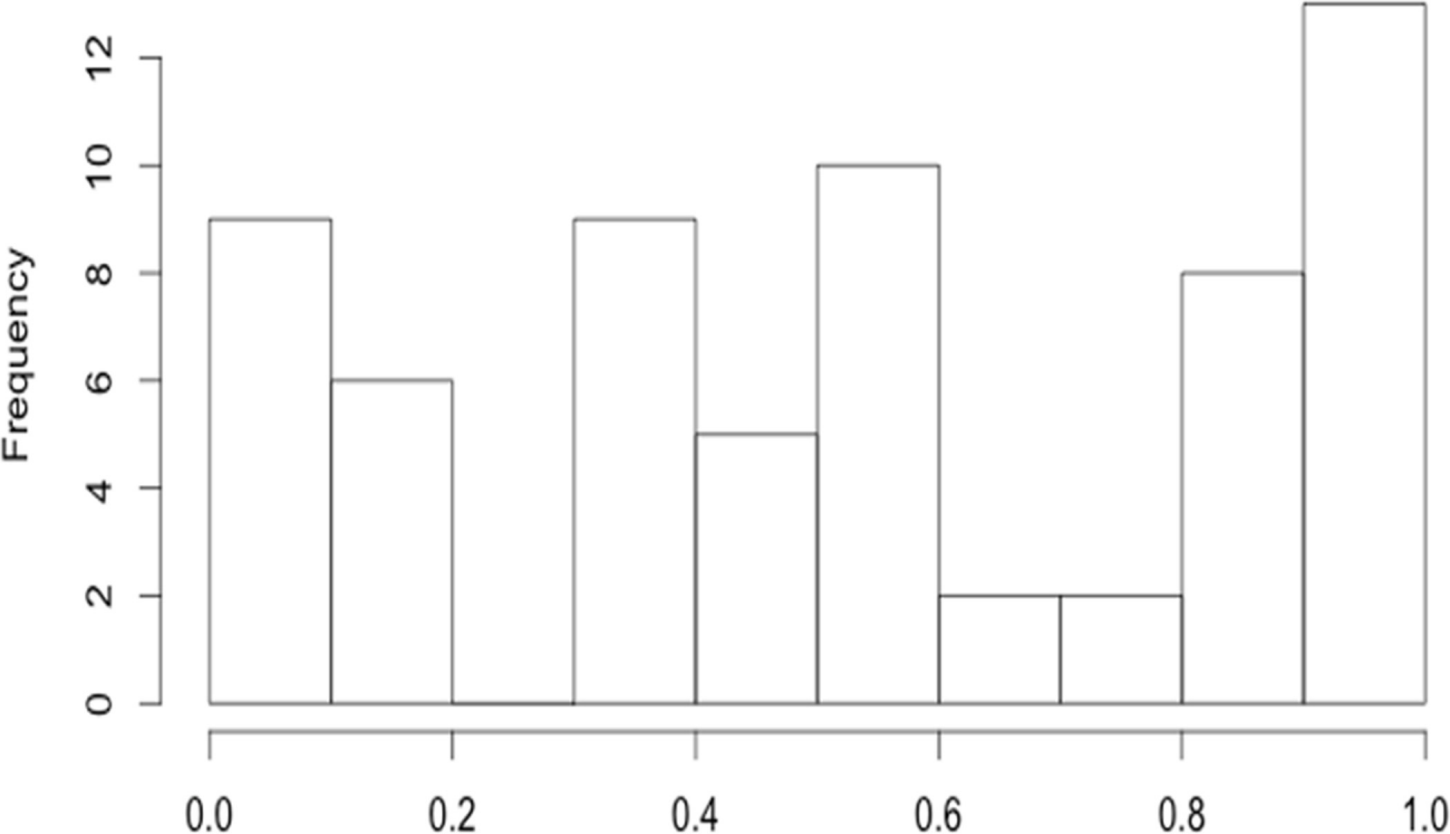
Histogram of missing rates of the 64 top-level UPDRS variables (median missing rate ∼ 0.5).

The PPMI clinical data includes cognitive assessments (e.g., cognitive state, decline, functional impairment, diagnosis, level of education). The MDS-UPDRS data captures the severity of PD symptoms in different dimensions using a 0–4 Likert scale representing a range of normal (unaffected, 0) to severely affected (4). There are four separate UPDRS sections, **Table 1**. UPDRS measures reflect a caregiver questionnaire interview and clinical observation. In our work, Part IV was not included in the analysis due to insufficient data. **Fig 4**shows the missingness distribution of the key UPDRS variables.

**Table 1:** Summary of UPDRS ratings.

UPDRS ratings		Missing
**Part I**	Uses 13 questions to measures non-motor aspects of experiences of daily living. It has two parts—Part 1A (neuropsychiatric symptoms) and Part IB (non-motor symptoms)	47.5%
**Part II**	Captures motor aspects of experiences of daily living (dressing, hygiene, tremor, freezing)	47.6%
**Part III**	Neurological motor examination (speech, rigidity, movement of extremities, and posture)	48.2%
**Part IV**	Motor complications (dyskinesia and motor fluctuations)	80.7%

Three cohorts of subjects were used: Group 1 = {*de novo* PD Subjects with a diagnosis of PD for two years or less who are not taking PD medications}, *N*_1_ = 263; Group 2 = {PD Subjects with Scans without Evidence of a Dopaminergic Deficit (SWEDD)}, *N*_2_ = 40; Group 3 = {Control Subjects without PD who are 30 years or older and who do not have a first degree blood relative with PD}, *N*_3_ = 127. The longitudinal PPMI dataset (e.g., clinical, biological and imaging data) was collected at PPMI clinical sites at screening, baseline (time = 0), 12, 24, and 48 month follow-ups. In the supplementary materials, we include a complete list of raw and derived data elements used in our analytics (*Data-elements*, *meta-data*, *complete aggregated datasets*, ***Data A in S1 File***).

### Data management

University of Michigan Institutional Review Board (IRB) reviewed and approved this study. Written informed consent was not required by individual study participants as the PPMI initiative manages the anonymization and de-identification and processing of patient records/information prior to data distribution and analysis. All PPMI data components were downloaded separately from the PPMI repository into a secure computational server managed by the Statistics Online Computational Resource (SOCR) [37–39]. Data formats included text, tabular, binary and ASCII files containing raw imaging, genetics, clinical and meta-data. According to meta-data and de-identified unique subject IDs, in-house scripts were implemented for data fusion and linking data elements from different sources. Inconsistencies and inhomogeneities were resolved *ad hoc* to ensure that the aggregated data were harmonized enabling subsequent multivariate imputation (assuming data missing at random). Extreme outliers were identified and corrected, as appropriate, by either imputing the erroneous values, replacing them with a linear model prediction for the corresponding cohort, or removing the case from the study. Intermediate results from pipeline workflow, R-scripts and Graphical user-interface tools were temporarily stored to complete the testing, validation and verification processes. As a major focus of the study was the supervised classification of PD patients and asymptomatic controls where patients outnumbered controls 3:1, we used group size rebalancing protocols to reduce potential bias and limit spurious effects due to cohort sample-size variations [40, 41]. The R package SMOTE (Synthetic Minority Over-sampling TEchnique) [42] enables learning from imbalanced datasets by creating synthetic instances of the under sampled class (e.g., controls). Most of our cohort comparisons were done on stratified data–by cohort-definition (HC vs PDs, or HC vs. Patients (PDs + SWEDDs)) and by cohort-size (raw unbalanced comparisons, or comparisons of SMOTE-rebalanced group sizes).

### Imputation

Depending on the cause of missingness in the data three alternative strategies for data imputation were utilized. In general, cases (subjects) or data elements (variables) with < 50% completeness were excluded from the study. Multivariate multiple imputation [43] was applied for missing data that involved data-simulation to replace each missing data point with a set of *m* > 1 *plausible* values drawn from (estimated) predictive distribution (univariate or joint multivariate). As the imputed values do not introduce biases, the result of multivariate multiple imputation enables the analysis of a complete dataset that has the same joint distribution of the original data (assuming missingness is at random). Scientific inference based on the imputed data (e.g., estimates of standard errors, p-values, likelihoods) is valid because it incorporates uncertainty due to the missing data and because it relies on efficient estimators. The efficiency of an estimator represents the minimum possible variance for the estimator divided by its actual variance and directly affects the subsequent statistical inference. The efficiency of imputation-based estimators is ${\left(1+\frac{\gamma }{m}\right)}^{-1}$, where *m* and *γ* represent the fraction of missing information and the number of imputations, respectively [44]. For example, for 50% missing information (*γ* = 0.5), a high rate of incompleteness, and *m* = 5 imputations, we expect to achieve 91% efficiency (suggesting that the variances of these estimators are close to minimum, efficiency ∼ 1). In our experiments, we used the R-packages MICE [45] and MI [46] to perform *m* = 5 imputations, as necessary.

The longitudinal analyses required imputing the missing neuroimaging data. This was accomplished by computing two separate regression models (patients and controls, separately) for predicting the baseline to follow-up relationships, $V_{i}^{Month\_24}∼V_{i}^{Baseline}$, for each ROI-metric pair (denoted here as a variable, *V*_*i*_), using the available data. This was necessary as not all subjects (controls or patients) had repeated/longitudinal imaging data, and some had incongruent records, e.g., 6, 18, 24, or 36 month data. Thus, we imputed the missing imaging data for subjects with incomplete data using the linear model corresponding to control or patient cohort.

### Predictive Analytics

We used model-based and model-free approaches for predictive analytics. The model-based approaches included generalized linear models (GLM) [47], mixed effect modeling with repeated measurements (MMRM) [48, 49], change-based models [50], and generalized estimating equations (GEE) [51]. The model-free predictive analytics involved forecasting [52, 53], classification [54], and data mining [55]. The specific model-free methods we tested include AdaBoost [56], support vector machine (SVM) [57], Naïve Bayes [58], Decision Tree [59], KNN [60], and K-Means [61] classifiers. Both types of approaches (model-based or model-free) facilitate classification, prediction, and outcome forecasting (e.g., disease state) using new or *testing* data containing the same clinical, demographic, imaging and phenotypic data elements.

The model-based analyses required preprocessing to extract critical features from the large and complex integrated dataset. This (unsupervised) feature-selection was accomplished using the R-package CARET (classification and regression training) [62], which ranks all data elements included in the aggregated collection. This variable ranking is based on a cost function that is determined by a training set of an SVM with a linear kernel. This score is used iteratively (until certain classification accuracy is reached) to drop features with lower ranks and effectively sift out the most relevant features that are ultimately included in the final feature set of a user-specified dimension.

Our change-based models use relative-change, instead of raw-score or average-score across time, of all longitudinal variables involved in the prediction modeling. For instance, we used the following formula to compute the relative-change (Δ): $\Delta_{U_{i}}=\frac{U_{i}^{Month\_24}-U_{i}^{baseline}}{U_{i}^{baseline}}$, for each subject’s UPDRS score (*U*_*i*_). Our confirmatory analyses used specific imaging variables previously implicated in Parkinson’s disease. These included regions of interest (ROIs), like bilateral superior parietal gyrus, putamen, and caudate [63, 64], and *average-area* and *volume* as ROI morphometry metrics [65, 66], which are less variable, but also less sensitive compared to the more exotic shape morphometry measures like fractal dimension, curvedness, or shape index [33].

### Validation

**Fig 5:**
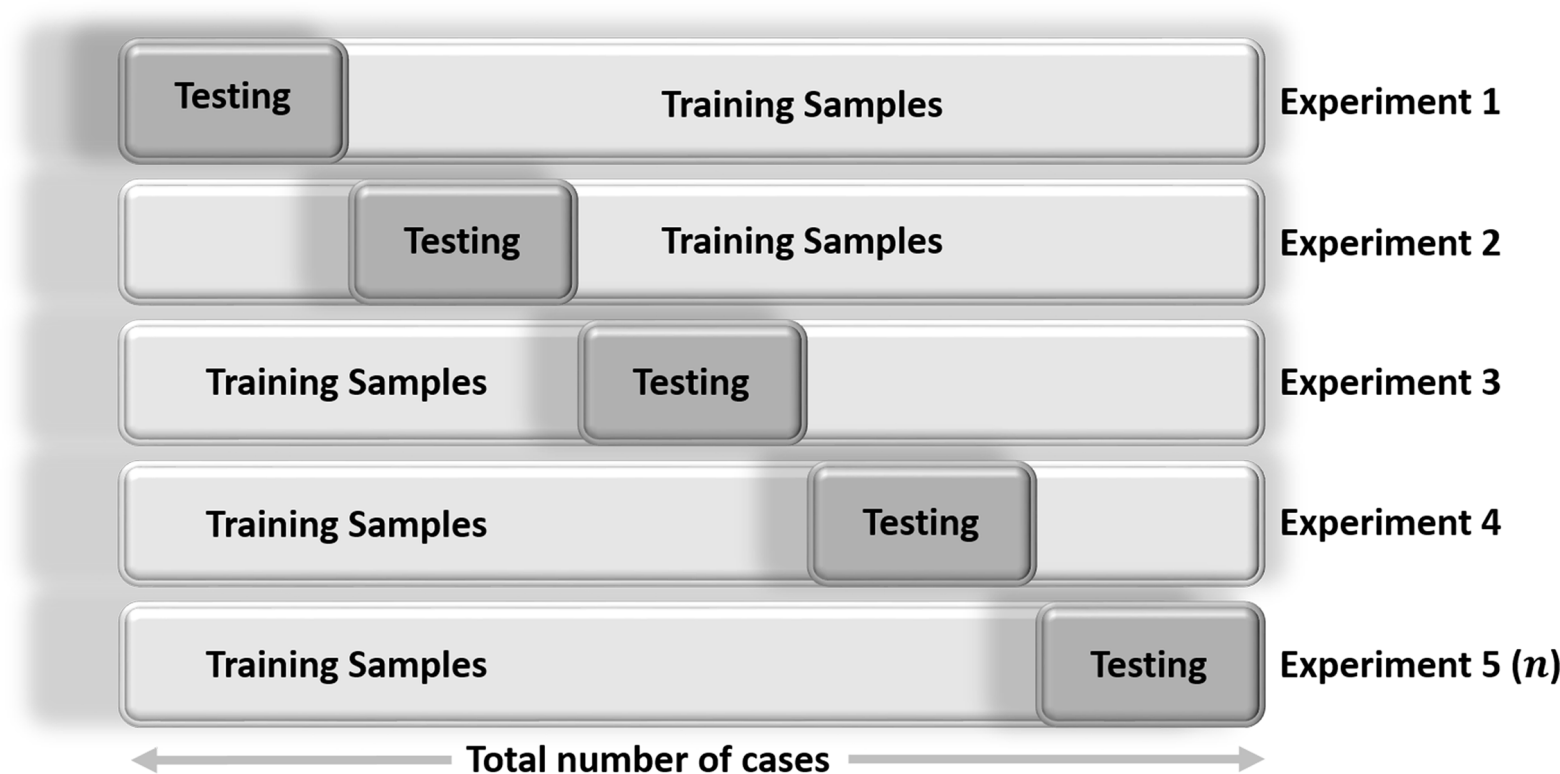
Schematic of iterative data splitting statistical n-fold cross-validation protocol.

In addition to optimizing the accuracy of a learning-based classifier on a training dataset, one needs to estimate its expected predictive performance using prospective (testing) data. Optimal data classification depends on many things including the choice of a classifier, model selection, the estimates of the bias, and the precision of the results. There is often a tradeoff between absolute prediction accuracy (precision) using training data with a classification reliability, as the desired high-precision (low bias) and low variance (high reliability) may not be simultaneously attainable [67, 68]. Statistical *n* -fold cross-validation [69, 70] is an alternative strategy for validating an estimate, a classification or a product without the need for a completely new prospective dataset where the predicted estimate can be tested against a gold-standard. Cross-validation relies on an *n* -fold partition of the available (retrospective) dataset. For each of *n* experiments, the algorithm uses *n* – 1 folds for *training* the learning technique and the last fold of the data for *testing* its accuracy. **Fig 5**shows a schematic of this *n* = 5 fold cross validation protocol. A random subsampling splits the entire dataset into *n* samples of equal size. For each data split we retrain the gold-standard classifier with the training examples and then estimate the error, *E*_*i*_ (predicted vs. gold-standard), using the test cases. The final (true) error estimate of the classification is obtained by averaging of the individual error estimates: $E=\frac{1}{n}\sum_{i=1}^{n}E_{i}$. Note that all the cases in the dataset are eventually used for both training and testing in the iterative *n* -fold cross-validation protocol. Larger *n* leads to large computational complexity and yields more accurate classification estimates (low bias), however, its reliability may be poor (variance of the true error rate may be larger). On the other hand, fewer number of folds leads to computationally attractive solutions with high reliability (low estimator variance), however, the bias of the estimator will be substantial (potentially high bias). In practice, we used the R package *crossval* (http://cran.r-project.org/package=crossval) with *n* = 5 for the number of cross-validation folds.

### Scientific Inference

Scientific inference (aka inferential reasoning) is a rational process linking core principles (knowledge), observed phenomena (data), and analytic representations (models), and can be conducted *deductively* or *inductively* [71]. Deductive scientific inference relies on formulating a hypothesis of system behavior *a priori*, and then testing the hypothesis’ validity. Thus, deductive scientific inference is objective, but it restricts our knowledge within the limited scope of the *a priori* hypotheses. Big Data inference frequently encompasses broad, diverse and novel discoveries that may or may not be anticipated, and so deductive approaches are overly restrictive in this context. The alternative, inductive scientific inference, depends upon the observed data itself to derive or suggest the most plausible conclusion, relation or pattern present in the phenomenon of interest. Evidence-based inductive reasoning subjectively transforms observations into a (forecasted) truth. Hence, inductive inference about prospective, complementary or unobserved states of the process has broader scope than the observed data used to derive these conclusions. Although inductive inference may be used to inform exploratory analytics (formulating new hypotheses, acquiring new knowledge, and predicting the state of new or partial data), it does not by itself support confirmatory analytics (e.g., making conclusive statements about the process, or teasing out causal effects). Our inductive approach to Big Data analytics provides more flexibility in a broader scope of inquiries, but additional validation and replication using other datasets are warranted. This approach is more suitable for large, complex and heterogeneous data. It provides the means of differentiating, classifying and predicting outcomes (e.g., diagnosis) by obtaining efficient data-driven estimates of likelihoods, probabilities and parameters describing the joint variability of the entire dataset.

## Results

### Data assembly

**Table 2**summarizes the methods, sample-sizes and characteristics of the main data ensembles used to test the model-based and model-free analytic approaches.

**Table 2:** Outline of the core study designs and data characteristics.

Inference Type	Methods	Comments on Data Restrictions	# of Cases
**Model-based or model-free inference**	Change-model (HC vs. PD or PD+SWEDD) GLM, MMRM or classification	Imaging data limited to bilateral surface-area, curvature, shape-index, curvedness and volume for the insular cortex and the cingulate gyrus	423
**Average between cohort differences**	GEE	UPDRS data limited to the following top-level variables: Part_I_Summary, Part_II_Patient_Questionnaire_Summary, Part_III_Summary, X_Assessment_Non.Motor_Epworth_Sleepiness_Scale_Summary, X_Assessment_Non.Motor_Geriatric_Depression_Scale_GDS_Short_Summary	406
**Model-free**	Various classifiers	(Raw) unbalanced and rebalanced (SMOTE) groups, all data elements with and without UPDRS (to contrast the power of no-UPDRS predictions)	423

Legend: HC = healthy controls; PD+SWEDD = (pooled cohort) Parkinson’s Disease and scans without evidence of dopaminergic deficit; GLM = generalized linear model; MMRM = Mixed-effect Model Repeat Measurement; GEE = generalized estimating equation; UPDRS = unified Parkinson's disease rating scale.

### Exploratory Data Analytics

**Tables 3**and **4**show the distributions of genders and their differences across the three research cohorts (HC, PD, SWEDD) indicating that there were not substantial gender deviations.

**Table 3:** Gender Distributions.

	Cohort			Total
Gender	HC	PD	SWEDD	
**F**	84	170	23	277
**M**	39	93	14	146
**Total**	123	263	37	423

**Table 4:** Gender Differences by Cohort.

Statistics	df	Value	Prob
***χ***^**2**^	2	0.7	0.705
Likelihood Ratio ***χ***^**2**^	2	1.1575	0.5606
Mantel-Haenszel	1	1.0453	0.3066

There were no significant *weight* (*F*_(2,427)_ = 0.06, *p* > 0.05) or *age* (*F*_(2,427)_ = 0.21, *p* > 0.05) differences among the cohorts. **Table 5**summarizes the statistical differences between the main 3 cohorts in this study in several demographic, genetic and clinical variables. Only the cognitive scores (COGSTATE, COGDECLN, COGDXCL) were significantly different between the HC, PD, SWEDD groups.

**Table 5:** Between-cohort differences in some demographic, genetic and clinical variables.

Variable	Categories/Classes	HC	PD	SWEDD	Exact Fisher's Test (p-value)
**sex**	1	84	170	23	0.711
	2	39	93	14	
**chr12_rs34637584_GT**	0	123	259	37	0.523
	1	0	4	0	
**chr17_rs11868035_GT**	0	67	127	15	0.189
	1	40	107	20	
	2	16	29	2	
**chr17_rs11012_GT**	0	84	176	28	0.733
	1	37	78	8	
	2	2	9	1	
**chr17_rs393152_GT**	0	77	160	26	0.904
	1	40	89	10	
	2	6	14	1	
**chr17_rs12185268_GT**	0	80	163	26	0.906
	1	39	88	10	
	2	4	12	1	
**chr17_rs199533_GT**	0	82	167	26	0.779
	1	39	86	10	
	2	2	10	1	
**COGSTATE**	Dementia (PDD)	0	5	0	**7.60E-08**
	Mild Cognitive Impairment (PD-MCI)	2	46	12	
	Normal Cognition (PD-NC)	121	212	25	
**COGDECLN**	No	121	230	31	**0.00025322**
	Yes	2	33	6	
**FNCDTCOG**	No	121	251	33	**0.04540301**
	Yes	2	12	4	
**COGDXCL**	10% - 49%	0	5	0	**0.000547062**
	50% - 89%	5	45	7	
	90% - 100%	118	213	30	

Our attempts to reduce the dimensionality of the complete dataset using classical methods were not very successful. A principal component analysis (PCA) [72] did not generate useful results to reduce the enormous dimensionality of the data (the first 20 components only explained 2/3 of the total variation). A Learning Vector Quantization (LVQ) [73] model was employed to rank the variable features by their importance, however, the result showed equivalent “importance” among most data elements (e.g., the top 10 features had similar “importance” as the next 10 features). The results of using Recursive Feature Elimination (RFE) [74] to build models, with cohort (research group) as the outcome required over 200 variables to get marginal discrimination power (accuracy = 0.6587). Hence, we did not pursue further dimensionality reduction.

### Model-based Analysis

We fit logistic regression models to predict the diagnostic outcome (either HC vs. PD or HC vs. PD+SWEDD). The resulting models had low predictive value and high Akaike Information Criterion (AIC) values [75], and remained largely unchanged when groups of covariates were excluded by reducing the model complexity. Next we tried feature selection using the hill-climbing search [76], followed by a generalized linear mixed model approach with genotype of rs11868035 (chr17) as a random effect. There were four significant diagnostic predictors (using a default false-positive rate of *α* = 0.05): *L_superior_parietal_gyrus_ComputeArea*, *L_superior_parietal_gyrus_Volume*, *R_superior_parietal_gyrus_AvgMeanCurvature*, and *R_superior_parietal_gyrus_Curvedness* (syntax encodes the labels of hemisphere, region and morphometry measure), all representing derived neuroimaging biomarkers. However, only the intercept remained significant by refitting the model including just these four predictors as fixed effects, and the same genotypic random effect. This indicates the unstable nature of the relation between response (diagnosis) and predictors (4 imaging covariates).

As another model-based prediction technique, generalized estimating equation (GEE) modeling offers an alternative to logistic regression. GEE models do not generate likelihood values for their estimates. Hence, the GEE results that can be directly compared to other models (e.g., using log-likelihood tests). Although GEE is useful as a confirmatory analysis of *average* population differences, it cannot be used to predict *individual* disease outcome using its parameter estimates, even though the latter are consistent with respect to alterations of the variable correlation structure. At the same time, GEE is computationally simpler (compared to GLM, GEE uses quasi-likelihood estimation, rather than maximum likelihood estimation, or ordinary least squares to estimate the model parameters) and does not require *a priori* knowledge of the joint multivariate distribution [77]. We used the R package *geeglm* (https://cran.r-project.org/web/packages/geepack/geepack.pdf) for our GEE model and the results are illustrated on **Table 6**.

**Table 6:** Most significant GEE model coefficients for covariates contributing to segregating mean cohort differences.

	Estimate	Std.err	Wald	Pr(>|W|)
**(Intercept)**	-7.2397	1.7924	16.32	**5.40E-05**
**chr12_rs34637584_GT**	-38.8454	1.6404	560.78	**<2.00E-16**
**chr17_rs393152_GT**	1.1731	0.5006	5.49	0.01911
**UPDRS_part_II**	-1.0406	0.1688	38.01	**7.00E-10**
**UPDRS_part_III**	-0.6709	0.0622	116.36	**<2.00E-16**

Using the longitudinal neuroimaging data (baseline, 12 and 24 follow ups), along with baseline UPDRS, we also fit GEE (handles missing data) and GLMM (requires balanced data) models (423 cases) to get a binary classification of subject diagnosis. **Table 7**shows the results of both experiments.

**Table 7:** GEE and GLMM predictive model summaries.

	GEE				GLMM				
	Estimate	Std.err	Wald	Pr(>|W|)	AIC	BIC	logLik	Deviance	Df.resid
(Intercept)	1.117257	0.723184	2.39	0.12237	193.8	380.9	-64.9	129.8	2530
L superior parietal gyrus ComputeArea	1.400402	1.497027	0.88	0.34955	**Fixed Effects:**			**Estimate**	**Pr(>|z|)**
L superior parietal gyrus Volume	-2.525902	1.359074	3.45	0.06309	(Intercept)			-7.82E+01	0.99904
R superior parietal gyrus ComputeArea	1.164381	0.976521	1.42	0.23311	L_superior_parietal_gyrus_ComputeArea			-1.94E+00	0.77698
R superior parietal gyrus Volume	-0.451386	1.007911	0.2	0.65427	L_superior_parietal_gyrus_Volume			2.34E+00	0.74594
L putamen ComputeArea	0.496793	0.713507	0.48	0.48626	R_superior_parietal_gyrus_ComputeArea			-1.36E-01	0.98267
L putamen Volume	-0.61937	0.83991	0.54	0.46086	R_superior_parietal_gyrus_Volume			1.31E+00	0.84431
R putamen Volume	0.836431	0.946288	0.78	0.37675	L_putamen_ComputeArea			-3.63E+00	0.52797
R putamen ShapeIndex	0.56228	0.354731	2.51	0.11295	L_putamen_Volume			2.84E+00	0.58167
L caudate ComputeArea	-0.413876	1.746183	0.06	0.81264	R_putamen_Volume			-2.95E-01	0.93113
L caudate Volume	1.580367	1.540474	1.05	0.30494	R_putamen_ShapeIndex			-2.06E-01	0.87846
R caudate ComputeArea	0.155381	1.410379	0.01	0.91227	L_caudate_ComputeArea			-6.77E+00	0.39352
R caudate Volume	-1.804119	1.502705	1.44	0.22991	L_caudate_Volume			3.38E+00	0.65557
**chr12 rs34637584 GT**	**35.800527**	**2.982947**	**144.04**	**<2.00E-16**	R_caudate_ComputeArea			5.30E+00	0.53451
chr17 rs11868035 GT	-0.716443	0.407688	3.09	0.07886	R_caudate_Volume			-2.08E+00	0.78918
chr17 rs11012 GT	-0.431071	0.891313	0.23	0.62864	chr12_rs34637584_GT			-4.51E+00	0.99272
**chr17 rs393152 GT**	**-1.230197**	**0.594327**	**4.28**	**0.03846**	chr17_rs11868035_GT			-4.92E-01	0.76636
**chr17 rs12185268 GT**	**2.556489**	**1.024266**	**6.23**	**0.01256**	chr17_rs11012_GT			7.34E-01	0.82913
chr17 rs199533 GT	-0.883482	0.889914	0.99	0.32082	chr17_rs393152_GT			2.92E+00	0.39461
**Sex**	**1.808176**	**0.577873**	**9.79**	**0.00175**	chr17_rs12185268_GT			3.02E+00	0.60026
Weight	0.473417	0.307536	2.37	0.12371	chr17_rs199533_GT			-2.90E+00	0.55589
**Age**	**-1.332754**	**0.330977**	**16.21**	**5.70E-05**	Sex			1.09E+00	0.68982
**UPDRS Part I Summary Score Baseline**	**-3.380284**	**0.72056**	**22.01**	**2.70E-06**	Weight			1.88E+00	0.16203
**UPDRS Part II Patient Questionnaire Summary Score Baseline**	**7.210265**	**1.560543**	**21.35**	**3.80E-06**	Age			1.83E+00	0.16687
**UPDRS Part III Summary Score Baseline**	**6.603029**	**2.144869**	**9.48**	**0.00208**	UPDRS_part_I			3.29E-01	0.84611
FID IID	0.000757	0.001309	0.33	0.56317	**UPDRS_part_II**			**-1.28E+01**	**0.0014**
**COGSTATE**	**-7.918928**	**1.672468**	**22.42**	**2.20E-06**	**UPDRS_part_III**			**-2.09E+01**	**0.00026**
**COGDECLN**	**-4.720291**	**1.398451**	**11.39**	**0.00074**	FID_IID			-1.60E-03	0.62892
**FNCDTCOG**	**32.085919**	**6.647111**	**23.3**	**1.40E-06**	COGSTATE			9.42E+00	0.23984
COGDXCL	-0.489857	1.434844	0.12	0.7328	COGDECLN			-4.48E+00	0.27759
EDUCYRS	0.07841	0.099566	0.62	0.43098	FNCDTCOG			8.71E+00	0.99989
					COGDXCL			-4.27E+00	0.39832
					EDUCYRS			3.68E-02	0.97715
**Estimated Correlation Parameters**									
		**Estimate**	**Std.err**						
**alpha**		**0.000159**	**0.000108**						
**Number of clusters:**	**423**	**Maximum Cluster**							
**size:**	**3**								

**Table 8**includes the results of a stepwise logistic regression model selection (using the *stepAIC* function in the MASS R package). The AIC of the final model was reduced to AIC = 170.57, compared to AIC = 212.45 of the initial complete GLM logistic regression model. The small, but negative, effect of Age, and the larger, but positive, effects of UPDRS (Part II and Part III summaries) indicate that an increase of age and decrease summary UPDRS scores are associated with presence (or higher severity) of Parkinson’s.

**Table 8:** Ultimate generalized linear logistic regression model (using step-wise AIC selection) illustrates that some UPDRS summaries (Parts II and III) along with Age play roles in explaining the diagnosis of participants.

	Estimate	Std.Dev.	Z	P(>|Z|)
**(Intercept)**	21.013	1796.808	0.01	0.9907
**L_caudate_Volume**	**1.574**	**0.633**	**2.49**	**0.0129**
**R_caudate_ComputeArea**	**-1.692**	**0.629**	**-2.69**	**0.0072**
**Sex**	0.993	0.627	1.58	0.1134
**Age**	**-1.079**	**0.355**	**-3.04**	**0.0024**
**UPDRS_Part_I_Summary_Score_Baseline**	-0.835	0.485	-1.72	0.0848
**UPDRS_Part_II_Patient_Questionnaire_Summary_Score_Baseline**	**7.92**	**1.791**	**4.42**	**9.80E-06**
**UPDRS_Part_III_Summary_Score_Baseline**	**5.25**	**0.901**	**5.83**	**5.70E-09**
**COGSTATEMild Cognitive Impairment (PD-MCI)**	-8.62	1796.809	0	0.9962
**COGSTATENormal Cognition (PD-NC)**	-13.982	1796.807	-0.01	0.9938
**FNCDTCOGYes**	-6.49	2.278	-2.85	0.0044

### Model-free Classification

**Table A** in S1 File includes the complete results from the machine learning based classification results using 24 different stratifications, 6(*methods*) × 2(*types*) × 2(*groups*), of the integrated PPMI dataset. Specifically, we tested *6* alternative classifiers including AdaBoost [56], SVM [57], Naïve Bayes [58], Decision Tree [59], KNN [60] and K-Means [61]. A summary of the best classification results is shown in **Table 9**. SVM and AdaBoost methods performed extremely well in automatically classifying new cases into controls or patients with simultaneously very low false-positive and false-negative predictions (high accuracy, precision and odds to detect Parkinson’s using the multi-source PPMI data). We computed a number of metrics to quantify the power of all classification results–false-positive (FP), true-positive (TP), true-negative (TN), false-negative (FN), accuracy, sensitivity, specificity, positive and negative predictive value (PPV/NPV), and log odds ratio (LOR).

**Table 9:** Best machine learning based classification results (according to average measures of 5-fold cross-validation).

Classifier	Cohorts	Balance	FP	TP	TN	FN	Accuracy	Sensitivity	Specificity	PPV	NPV	LOR
**adaboost**	PD vs. HC	balanced	0.2	72.2	98.2	1.6	0.98954704	0.97831978	0.99796748	0.99723757	0.98396794	10.0058805
**adaboost**	PD+SWEDD vs. HC	balanced	1.2	72	97.2	1.8	0.9825784	0.97560976	0.98780488	0.98360656	0.98181818	8.08332861
**adaboost**	PD vs. HC	unbalanced	0.4	22.4	52.2	2.2	0.96632124	0.91056911	0.99239544	0.98245614	0.95955882	7.19197683
**svm**	PD vs. HC	balanced	2	69.4	96.4	4.4	0.96283391	0.9403794	0.9796748	0.9719888	0.95634921	6.63364135
**adaboost**	PD+SWEDD vs. HC	unbalanced	0.8	22.4	59.2	2.2	0.96453901	0.91056911	0.98666667	0.96551724	0.96416938	6.62466869
**svm**	PD+SWEDD vs. HC	balanced	2.8	67.2	95.6	6.6	0.94541231	0.91056911	0.97154472	0.96	0.93542074	5.851157

### Prediction

**Fig 6:**
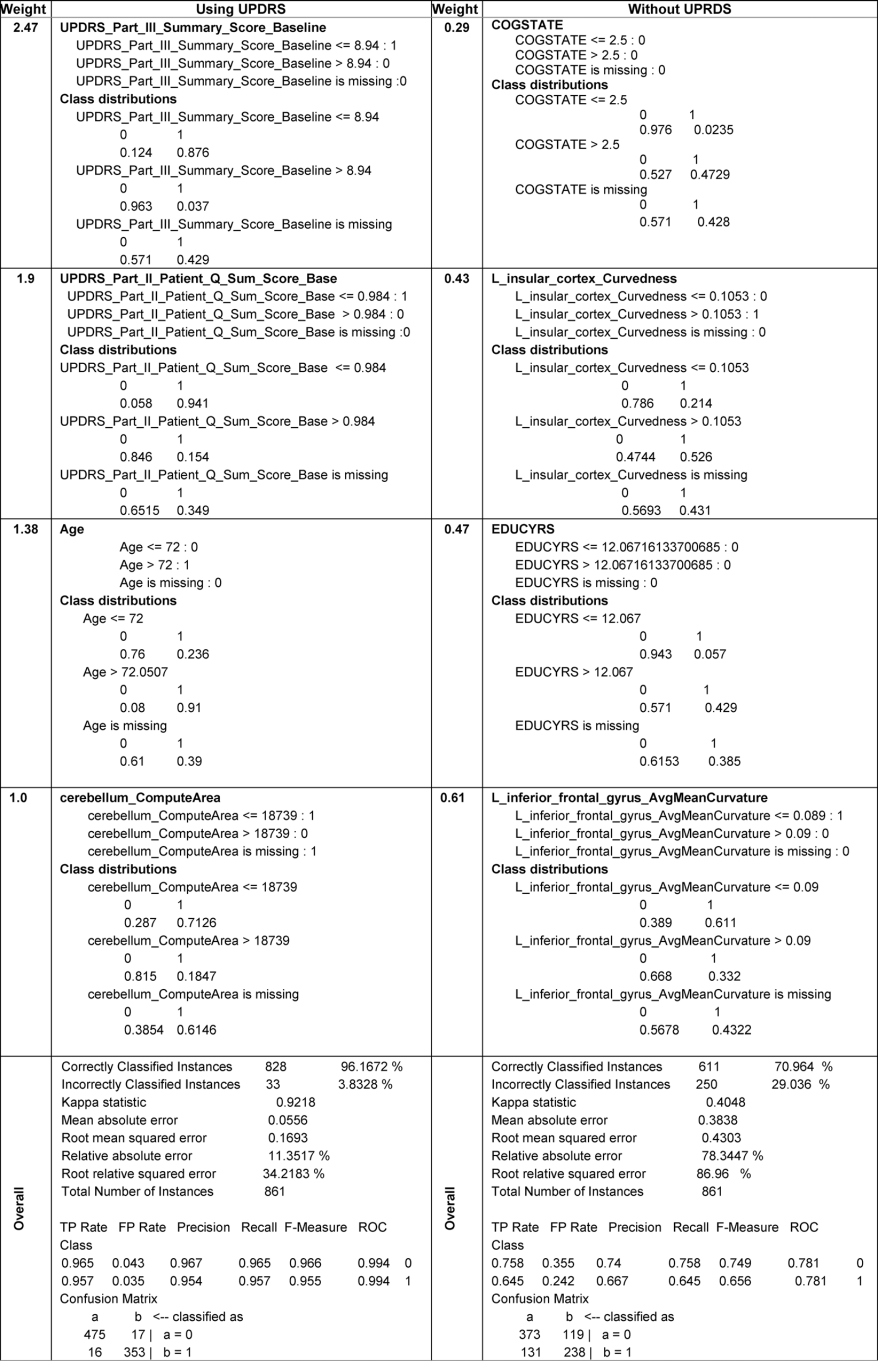
Fragments of the analytical representations and class distributions of the AdaBoost classifier (complete details are in Data B in S1 File, *AdaBoost Classifier model (based on RWeka)*) generated by the first step (predictive *analysis*).

In the supplementary materials we provide the complete descriptions of the model-based and model-free classification approaches (*Classification Results*, **Table A** and **Fig A** in **S1 File**). **Fig 6**contains a summary illustrating the format of the predictive classification methods explicitly identifying the critical data elements (with high weight coefficients) and their individual explanatory power (binary class distributions), see also **Data B** in **S1 File** (Classifier information of AdaBoost). We have developed *analysis* and *synthesis* R-scripts to invoke, save and test the SVM and AdaBoost classifiers. The analysis script estimates the classification parameters and saves their analytic representations as objects or files. The synthesis script loads the classifier representation generated by the analysis script, imports prospective data (new subjects), and generates class membership predictions (e.g., forecast subject diagnosis), which can be evaluated on an individual or a cohort level.

**Fig 7:**
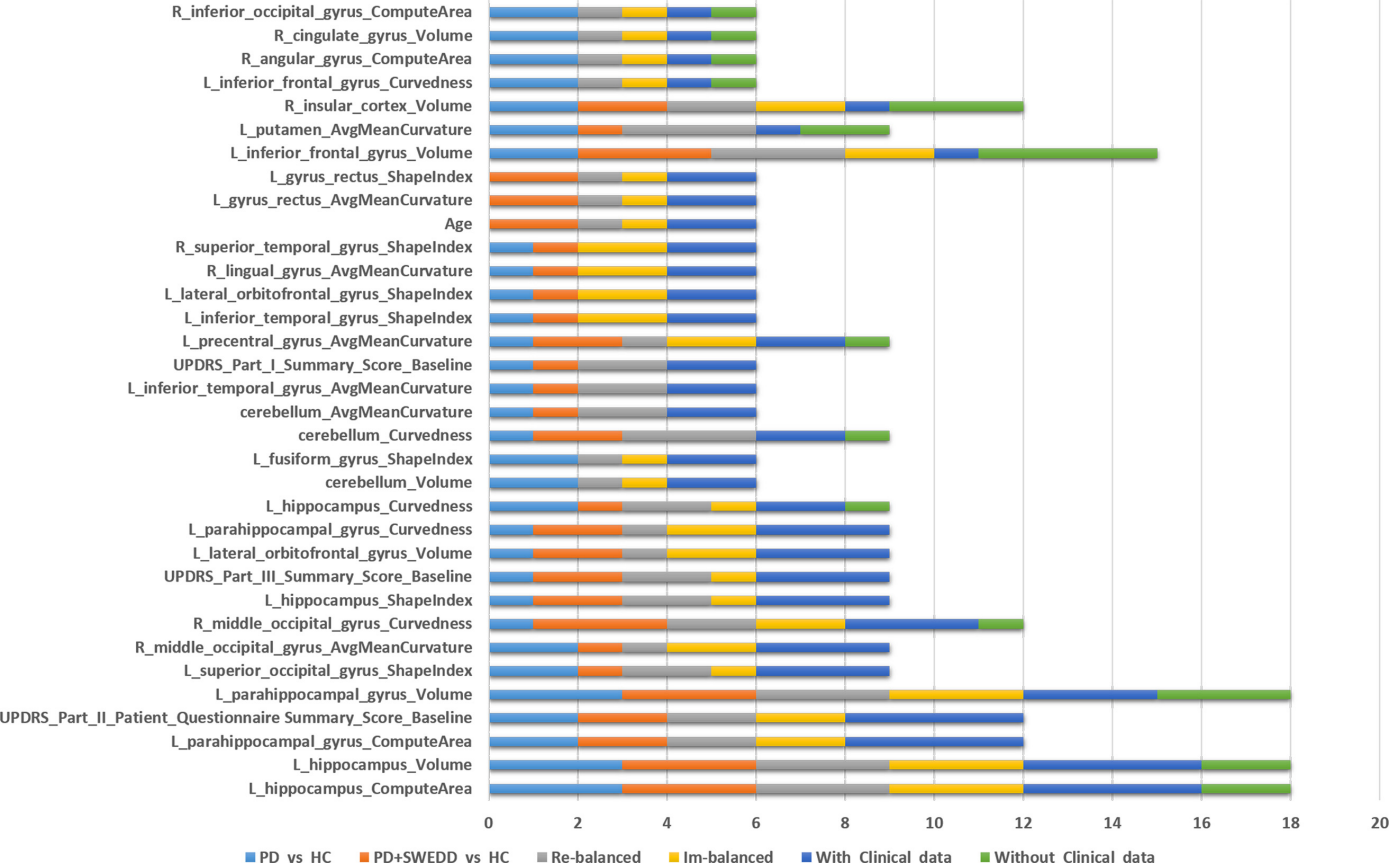
(Partial) Varplots illustrating some of the critical predictive data elements for AdaBoost classifier predicting HC vs. (PD+SWEDD).

The supplementary materials (S1 File, *AdaBoost Model*) include the varplots, representing the impact of most influential predictors, for all eight studies– 2(variable collections), 2(patient cohorts), and 2(cohort balance strategies). **Fig 7**includes several illustrative examples (additional varplots are included in **Fig B** to **Fig I in S1 File**).

### Statistical Validation

Using the R package *crossval* we performed *n*-fold cross validations with *n* = 5 and number of repetitions 1 ≤ B ≤ 5. As the outcome variable (diagnosis) was a factor, balanced sampling was used where in each iteration all categories are represented proportionally. To enable the predictive analytics, this statistical validation reports the stochastic estimates for each cross validation run, averages over all cross validation runs, and their corresponding standard errors. All results reported in **Table 9**and **Table A** and **Data B** in **S1 File**, include the average measures of 5-fold cross-validation. Thus, the results are expected to be reproducible on prospective data as statistical validation iteratively generates many random subsamples of the data where each subject serves independently as a testing (for the analytic learning process) or a training (for the synthesis prediction process) case in our classification protocol.

## Discussion

This study utilized translational techniques to harmonize, aggregate, process and analyze complex multisource imaging, genetics, clinical, and demographic data. Our ultimate aim was to develop classification and predictive models for Parkinson’s disease using PPMI data. At this point in time, the fundamental data science theory is not developed to allow us to jointly model the initial dataset holistically and obtain predictive analytics based on the entire raw data. Thus, our approach is based on analyzing the different types of data (e.g., genomics, imaging, clinical) independently and extracting derived biomarkers for each class of data elements. Then, we fused these derived biomarkers to generate homologous multidimensional signature vectors for each case (participating subject), fitted model-based and trained model-free classifiers, and finally internally validated the automated diagnostic labels using statistical n-fold cross validation. This approached allowed us to circumvent sampling incongruency, incomplete data elements, and complexities involved in the representation of multisource data.

Our results suggest that, at least in this situation of discriminating between Parkinson’s patients and asymptomatic controls, the machine learning based model-free techniques generate more consistent and accurate diagnostic classifications and disease predictions compared to model-based methods. SVM and AdaBoost generated high-fidelity predictive results automatically identifying participants with neurodegenerative disorders based on multifaceted data. This study demonstrates that extreme misbalances, in training data, between cohorts sample sizes may impact substantially the classification results. Our approach involved developing an end-to-end reproducible protocol based on open-source software and computational services (e.g., R, Pipeline, SOCR). This pipeline workflow protocol is shared with the community to facilitate external validation, promote open-science, and allow for collaborative contributions to modify, expand or merge this protocol with other atomic tools, web-services, or more elaborate study designs.

Our key findings suggest that a blend of clinical (e.g., Unified Parkinson's Disease Rating Scale (UPDRS) scores), demographic (e.g., age), genetics (e.g., rs34637584, chr12), and derived neuroimaging biomarker (e.g., cerebellum shape index) data contributed to the predictive analytics and diagnostic forecasting of Parkinson’s disease. A very interesting finding was that the classification results were significantly improved when we used the SMOTE rebalanced data, compared to the raw imbalanced cohort sizes. For example, when predicting HC vs. PD, the SVM classifier for the rebalanced cohorts yielded *accuracy* = 0.96283391, *sensitivity* = 0.9403794 and *specificity* = 0.9796748. Whereas for the imbalanced (native group sizes), the SVM reliability was much lower, *accuracy* = 0.75906736, *sensitivity* = 0.28455285, *specificity* = 0.98098859. Similarly, and as expected, inclusion of UPDRS variables into the prediction analytics significantly enhanced the classification reliability during the prediction synthesis phase. When UPRDS data were included in the classification analysis they dominated the variable rankings, as their weight coefficients were larger indicating their highly predictive PD-diagnostic characteristics, see the supplementary materials for details (**Data B** in S1 File, *AdaBoost Model*). One example of a single run of the AdaBoost classifier shows the significant contribution of UPDRS data on accuracy of the model-free classification results, **Table 10**. These results indicate that other (non-UPDRS) data elements may be associated with PD and more research would be necessary to further improve the machine learning classifiers (in terms of their precision and reliability). Another possible future direction is to examine the differences between PD and SWEDD patients, early vs. late onset PD, and other cohort stratifications based on disease stage, motor function, or health-related quality of life [78]. The timing of the observed imaging changes, relative to the onset of disease symptoms, is a key point for further investigation. In addition to its usefulness as a predictive diagnostic tool, this method may be included as a potential biomarker, itself, when changes of non-UPDRS variables are observed following the symptom manifestations.

**Table 10:** Example of the impact of including/excluding UPDRS data on the accuracy of the AdaBoost classification.

Dataset	sensitivity	specificity	accuracy
**no UPDRS data**	0.871794872	0.25	0.8203125
**including UPDRS**	1.0	0.96875	0.990024938

Our results illustrates that mixtures of raw data elements and derived biomedical markers provide a significant power to classify subject phenotypes (e.g., clinical diagnosis), even in situations where the data elements forming the basis of the clinical phenotypes are excluded (see **Tables 9 and 10**). In addition to the importance of classical clinical variables, e.g., UPDRS scores, several derived morphometric imaging measures associated with regional brain parcellations, that have not been previously broadly studied or reported, show in our analyses as important predictors of PD, e.g., cerebellum curvature, putamen, inferior frontal gyrus, and brain stem volume. At the same time, other cognitive (e.g., COGSCORE), demographic (e.g., EDUCYRS), and genotypic (e.g., chr12_rs34637584_GT), variables played a lesser role in the machine learning based PD diagnostic classification.

Some brain regions, like the left hippocampus, appear as major predictive factors in segregating both the pure PDs and PD+SWEDD cohorts from HC, in both raw imbalanced group sizes, as well as in rebalanced cohorts. However, the accuracy of the patient-control classification was much higher in the statistically rebalanced samples indicating a potential for significant sample-size effects. As expected, inclusion of the most relevant clinical biomarkers (UPDRS scores) into the training of all classification methods substantially increased the accuracy of the corresponding diagnostic prediction assessed using the testing data, **Table A** and **Fig A in S1 File**.

Previous studies of predicting Parkinson’s diagnosis using machine learning, classification and data-mining methods have reported 70–90% sensitivity in predicting the diagnosis [79] using (1) stepwise logistic model classification based on olfactory function, genetic risk, family history, and demographic variables [80]; (2) enhanced probabilistic neural networks based on motor, non-motor, and neuroimaging data [81]; (3) machine learning methods based on voice acoustics measures of vowel articulation (e.g., ratios of the first and second formants of the corner vowels *i*, *u*, and *a*, reflecting the movements of the tongue, lips, and jaw) [82], fuzzy *k*-nearest neighbor and SVM methods based on clinical, vocal and facial data [79], SVM diagnostic classification based on proteomic profiling, mass spectrometry analysis of cerebrospinal fluid peptides/proteins [83], Fisher-discriminant ratio feature extraction and least squares SVM using brain imaging and demographic data [84], random forest classification based on gait and clinical characteristics (e.g., age, height, body mass, and gender, walking speed, pathological features) [85], booststrap resampling with relevance vector machine (RVM) for multiclass classification using neuroimaging and clinical data [86].

The novelty of our approach is rooted in the integration of heterogeneous multisource imaging, clinical, genetics and phenotypic data, the realization of the importance of rebalancing imbalanced cohorts, the evaluation of a wide class of diagnostic prediction techniques, and the construction of an end-to-end protocol for data preprocessing, harmonization, analysis, high-throughput analytics and classification. Testing, validation, modification and expansion of this pipeline protocol by the entire community will ultimately serve to validate the reported findings using new data. Most studies of complex human conditions demand a multispectral examination of environmental condition, (epi)genetic traits, clinical phenotypes, and other relevant information (e.g., pathological reports, imaging, tests). In the case of automatic classification of Parkinson’s disease, we demonstrated the importance of using such heterogeneous data to accurately predict the observed clinical phenotypes using machine learning strategies. The internal validation of our machine learning based classification of Parkinson’s patients suggests that this approach generates more reliable diagnostic labels than previously reported methods, as the accuracy, sensitivity and specificity of several classifiers exceed 95% for the rebalanced cohorts. As we report, and save, the resulting models, these classifiers can be applied directly to other datasets (with similar data elements), retrained and adapted to process data with substantially different structural elements, or used for variable selection in other prospective Parkinson’s disease studies.

## Conclusions

In this study, we constructed an end-to-end protocol for data synthesis and analysis, manipulation, fusion and aggregation, as well as processing and diagnostic forecasting. We used three stratification strategies to examine the effects of patient groupings, imbalances of cohort sizes, and the impact of UPDRS data on the reliability of the diagnostic classifications. Two designs comparing the asymptomatic HC to patients were employed–HC vs. PD (alone) or HC vs. (PD+SWEDD). The effects of cohort sizes were explored by separately modeling the default (unbalanced) cohorts and the balanced cohort sizes (using statistical sample-size rebalancing). Finally, we investigated the reliability of the classifiers to predict subject diagnosis with or without using the UPDRS variables. This was important, as clinical PD diagnosis is heavily impacted by the results of the UPDRS tests.

**Fig 8:**
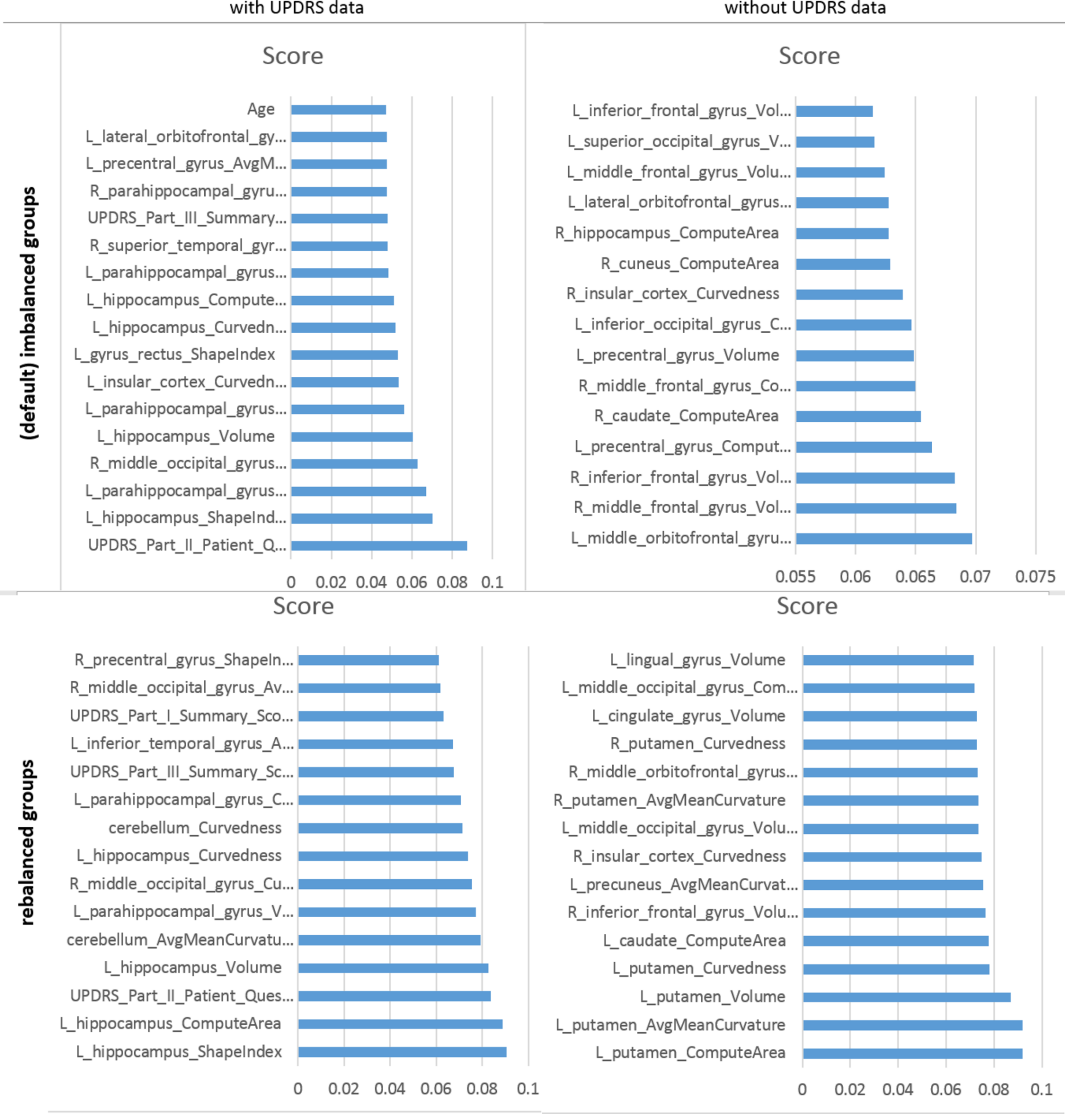
Frequency plot of data elements that appear as more reliable predictors of subject diagnosis, ranked by counts using rebalanced URPRS data (see Table B in S1 File for the complete numerical results).

Aside from the critical importance of UPDRS scores as the basis for non-invasive clinical diagnosis of Parkinson’s disease, there were three derived imaging biomarkers (paired hemispheric, region of interest, and morphometry-measure indices) that consistently stood out as important factors in the prediction of the final diagnostic outcome (across study-designs). These were *R_middle_orbitofrontal_gyrus_AvgMeanCurvature*, *L_supramarginal_gyrus_ShapeIndex*, and *L_superior_occipital_gyrus_AvgMeanCurvature*. From the demographic factors, *Age* showed a great consistency of impact in most experiments. Although we tried several alternative classifiers, AdaBoost consistently outperformed the others in most experiments. To illustrate the impact of different variables on the automated diagnostic predictions based on the AdaBoost machine learning classifier we tabulated the frequencies of data elements occurrence in the top-30 (most impactful) predictors for each of eight experiments. **Fig 8**and **Tables A** and **B in S1 File** show the frequency counts of how often each variable played a critical role (top-30 variables only) in the predictive analytics. Higher frequency counts indicate more significant overall impact. The eight experiments included comparing HC vs. patients (PD only, or PD+SWEDD), statistically rebalanced or imbalanced (default) cohort sizes, and the use of clinical data (UPDRS, Cognitive values). For a given experimental condition (e.g., comparing HC vs. PD), there are four sub-experiments (one for each balancing strategy and with/without use of clinical data). Thus, each data element takes on values between 0 (low impact) and 4 (high overall impact). Clearly there are derived neuroimaging biomarkers, UPDRS scores and age that appear to be more consistent across different study designs, which may indicate their overall importance in the predictive diagnostic analytics.

### Prospective Validation

To complement our statistical validation and confirm the reproducibility of these findings, new test data may be processed through the same methods/tools for predictive PD Big Data analytics. For instance, the Alzheimer’s Disease Neuroimaging Initiative (ADNI) [87] and the Global Alzheimer's Association Interactive Network (GAAIN) Consortium [88] have longitudinal neuroimaging data including patients that developed PD during their monitoring. Other multi-source, complex and heterogeneous data of neurodegenerative disorders containing at-risk populations (e.g., patients with REM sleep behavior disorder some of which eventually develop Parkinson’s [89]) may also be useful for a subsequent data-driven validation of these models. Additional PD biospecimen, pathological, and amyloid-based imaging data can add to the pool of complex data we used in this study to provide complementary biomolecular information enhancing the detection, tracking, and forecasting of the disease. In this study, the model-free methods underwent cross-validation as an internal validation strategy. This may potentially increase the risk for over-fitting and false discovery due to independent applications of 6 techniques. Ultimately, an external validation, based on an independent sample or prospective data, will be valuable to confirm our findings.

Handling incomplete data is always challenging, especially in Big Data studies, because the cause of missingness and the approach to imputing the data may potentially introduce bias in the results. In this study, we only used data that was mostly complete (50% ≤ observed rate ≤ 100%) and imputed the missing data elements, see **Table 1**and **Fig 4**. In our preprocessing, we did examine the missingness patterns in the data, which did not indicate the presence of strong non-random missing drivers. To validate the imputation process, we compared the distributions of the native data elements to their corresponding imputed counterparts and examined the convergence of the multiple imputation protocol by comparing the variance between chains (*B*) to the variance within chains (*W*). To control the number of iterations within imputation chains, we used the standard diagnostic criterion in terms of the potential scale reduction factor, $\hat{R}=\sqrt{\frac{P}{W}}\leq 1.1$, where the marginal posterior variance, $P=\frac{n-1}{n}W+\frac{1}{n}B$, represents a weighted average of the between and within chain variability [90]. In our model-free analyses, we did not rank the variables in terms of their importance prior to preprocessing. The variable impact on classifying patients and controls was only assessed at the end of the predictive analytics, when we reported their importance into the classification labeling (e.g., **Fig 6**, **Table 10**). Indeed, a deeper examination may be conducted into the relation between variable missingness and impact.

### Potential Limitations

The impact of extreme data heterogeneity, type and format complexity, non-random patterns of missingness, or substantial confounding between multiple covariates may present challenges and/or require substantial preliminary work to ensure sufficient starting congruency arrangement allowing the application of our protocol to new testing data archives. An appropriate computational platform, including functioning version of R 3.2.2, the Pipeline environment, SOCR libraries, and the suite of neuroimaging processing tools included in the Global Shape Analysis Pipeline workflow, is required to replicate these results or execute the same protocol in prospective studies. We do provide free access to this environment via the Big Data Discovery Science computational services at the Institute for Neuroimaging and Informatics (for account application see http://pipeline.loni.usc.edu and http://pipeline.loni.usc.edu/get-started/become-a-collaborator). Some of the imputation, rebalancing, or classification methods may fail when encountering points of singularity (e.g., negative Hessian matrices, unstable convergence, low-rank variance-covariance matrices). Resolving such cases is sometimes possible, but may require certain technical expertise, appropriate training, experimentation, deep dive into software documentation, or testing of alternative modules, software packages, or computational libraries mediating the failing processing step.

### Resource Availability

All raw PPMI data is available from the PPMI consortium (www.ppmi-info.org/data). The computational protocol, source-code, scripts, and derived data we generated as part of this study, along with the complete GSA pipeline workflow and corresponding R scripts, are available on GitHub BDDS (https://github.com/BD2K/BDDS) and PBDA (https://github.com/SOCR/PBDA) repositories.

Longitudinal UPDRS data may provide powerful mechanisms to diagnose PD, track its progression, and perhaps determine the efficacies of various medical treatments. However, missing, incomplete and incongruent Big Data, including UPDRS scores, present substantial analytic challenges. We have tested several strategies to deal with various Big Data barriers specifically involving PPMI demographics, clinical, genetics and neuroimaging data. There is a clear need to carry out deeper studies exploring the progression of PD across time, investigate techniques for subject-specific modeling using known cohort trends, replicate and expand previously reported findings, explore multinomial classification of PD phases of finely stratified cohorts (e.g., HC, PD, SWEDD), and confirm the statistical validation by testing the classifiers using prospective data (following an appropriate data-model harmonization). Our results demonstrate that model-free machine learning classification methods outperform model-based techniques in terms of the precision and reliability of predicting patient diagnosis in terms of complex and heterogeneous data.

## Supporting Information

S1 File (DOCX)
