# Supplementary material for: Predictive Big Data Analytics: A Study of Parkinson’s Disease Using Large, Complex, Heterogeneous, Incongruent, Multi-Source and Incomplete Observations
Source: PLoS One. 2016 Aug 5;11(8):e0157077. doi: 10.1371/journal.pone.0157077 (PMC4975403; doi:10.1371/journal.pone.0157077)
Supplement: S1 File — (DOCX) [file pone.0157077.s001.docx]

**Supplementary Data/Materials for PONE-D-15-42694.R1**

**S1 File**

**Predictive Big Data Analytics: A Study of Parkinson’s Disease using Large, Complex, Heterogeneous, Incongruent, Multi-source and Incomplete Observations**

Ivo D. Dinov ^1,5,8^, Ben Heavner ^2^, Ming Tang ^1^, Gustavo Glusman ^2^, Kyle Chard ^4^, Mike Darcy ^3^, Ravi Madduri ^4^, Judy Pa ^5^, Cathie Spino ^8^, Carl Kesselman ^3^, Ian Foster ^4^, Eric W. Deutsch ^2^, Nathan D. Price ^2^, John D. Van Horn ^5^, Joseph Ames ^5^,

Kristi Clark ^5^, Leroy Hood ^2^, Benjamin M. Hampstead ^6,7^, William Dauer ^8^, and Arthur W. Toga ^5^

^1^ Statistics Online Computational Resource, School of Nursing, Michigan Institute for Data Science,

University of Michigan, Ann Arbor, MI.

^2^ Institute for Systems Biology, Seattle, WA.

^3^ Information Sciences Institute, University of Southern California, Los Angeles, CA.

^4^ Computation Institute, University of Chicago and Argonne National Laboratory, Chicago, IL.

^5^ Stevens Neuroimaging and Informatics Institute, University of Southern California, Los Angeles, CA.

^6^ Department of Psychiatry and Michigan Alzheimer’s Disease Center, University of Michigan, Ann Arbor, MI.

^7^ Veterans Affairs Ann Arbor Healthcare System, Ann Arbor, MI.

^8^ Udall Center of Excellence for Parkinson’s Disease Research, University of Michigan, Ann Arbor, MI.

Contents

[S1 File: Appendix (Supplementary Materials) 3](#_Toc453418788)

[Pipeline workflow (XML/PIPE) 3](#_Toc453418789)

[Data A 3](#_Toc453418790)

[**Table A** 7](#_Toc453418791)

[**Fig A** 8](#_Toc453418792)

[Data B 9](#_Toc453418793)

[Data C 15](#_Toc453418794)

[Data D 21](#_Toc453418795)

[**Fig B** 22](#_Toc453418796)

[**Fig C** 22](#_Toc453418797)

[**Fig D** 23](#_Toc453418798)

[**Fig E** 23](#_Toc453418799)

[**Fig F** 24](#_Toc453418800)

[**Fig G** 24](#_Toc453418801)

[**Fig H** 25](#_Toc453418802)

[**Fig I** 25](#_Toc453418803)

[Table B 26](#_Toc453418804)

[Table C 30](#_Toc453418805)

[Table 5 31](#_Toc453418806)

[Table 7 32](#_Toc453418807)

[Table 10 34](#_Toc453418808)

[Figure 7 36](#_Toc453418809)

# S1 File: Appendix (Supplementary Materials)

### Pipeline workflow (XML/PIPE)

The global shape analysis pipeline workflow used to obtain the 280 ($2\times56\times5$) derived neuroimaging biomarkers from the structural MRI brain data for all subjects is attached as a portable XML/PIPE file. It is also available for hands-on testing using the USC Pipeline guest online services (<http://bit.ly/1DjhkG9>).

### Data A

Data-elements, meta-data, complete aggregated datasets. The list below includes all raw and derived data elements used in this study:

**Unique Subject Identifier**: FID_IID

**Derived Neuroimaging Biomarkers**: (28 regions, bilaterally (left and right hemispheres), 5 separate morphometry measures), see <https://wiki.loni.usc.edu/index.php/Global_Shape_Analysis_%28GSA%29_Workflow>:

L_insular_cortex_AvgMeanCurvature L_insular_cortex_ComputeArea L_insular_cortex_Volume L_insular_cortex_ShapeIndex L_insular_cortex_Curvedness R_insular_cortex_AvgMeanCurvature R_insular_cortex_ComputeArea R_insular_cortex_Volume R_insular_cortex_ShapeIndex R_insular_cortex_Curvedness L_cingulate_gyrus_AvgMeanCurvature L_cingulate_gyrus_ComputeArea L_cingulate_gyrus_Volume L_cingulate_gyrus_ShapeIndex L_cingulate_gyrus_Curvedness R_cingulate_gyrus_AvgMeanCurvature R_cingulate_gyrus_ComputeArea R_cingulate_gyrus_Volume R_cingulate_gyrus_ShapeIndex R_cingulate_gyrus_Curvedness L_caudate_AvgMeanCurvature L_caudate_ComputeArea L_caudate_Volume L_caudate_ShapeIndex L_caudate_Curvedness R_caudate_AvgMeanCurvature R_caudate_ComputeArea R_caudate_Volume R_caudate_ShapeIndex R_caudate_Curvedness L_putamen_AvgMeanCurvature L_putamen_ComputeArea L_putamen_Volume L_putamen_ShapeIndex L_putamen_Curvedness R_putamen_AvgMeanCurvature R_putamen_ComputeArea R_putamen_Volume R_putamen_ShapeIndex R_putamen_Curvedness L_hippocampus_AvgMeanCurvature L_hippocampus_ComputeArea L_hippocampus_Volume L_hippocampus_ShapeIndex L_hippocampus_Curvedness R_hippocampus_AvgMeanCurvature R_hippocampus_ComputeArea R_hippocampus_Volume R_hippocampus_ShapeIndex R_hippocampus_Curvedness cerebellum_AvgMeanCurvature cerebellum_ComputeArea cerebellum_Volume cerebellum_ShapeIndex cerebellum_Curvedness brainstem_AvgMeanCurvature brainstem_ComputeArea brainstem_Volume brainstem_ShapeIndex brainstem_Curvedness L_superior_frontal_gyrus_AvgMeanCurvature L_superior_frontal_gyrus_ComputeArea L_superior_frontal_gyrus_Volume L_superior_frontal_gyrus_ShapeIndex L_superior_frontal_gyrus_Curvedness R_superior_frontal_gyrus_AvgMeanCurvature R_superior_frontal_gyrus_ComputeArea R_superior_frontal_gyrus_Volume R_superior_frontal_gyrus_ShapeIndex R_superior_frontal_gyrus_Curvedness L_middle_frontal_gyrus_AvgMeanCurvature L_middle_frontal_gyrus_ComputeArea L_middle_frontal_gyrus_Volume L_middle_frontal_gyrus_ShapeIndex L_middle_frontal_gyrus_Curvedness R_middle_frontal_gyrus_AvgMeanCurvature R_middle_frontal_gyrus_ComputeArea R_middle_frontal_gyrus_Volume R_middle_frontal_gyrus_ShapeIndex R_middle_frontal_gyrus_Curvedness L_inferior_frontal_gyrus_AvgMeanCurvature L_inferior_frontal_gyrus_ComputeArea L_inferior_frontal_gyrus_Volume L_inferior_frontal_gyrus_ShapeIndex L_inferior_frontal_gyrus_Curvedness R_inferior_frontal_gyrus_AvgMeanCurvature R_inferior_frontal_gyrus_ComputeArea R_inferior_frontal_gyrus_Volume R_inferior_frontal_gyrus_ShapeIndex R_inferior_frontal_gyrus_Curvedness L_precentral_gyrus_AvgMeanCurvature L_precentral_gyrus_ComputeArea L_precentral_gyrus_Volume L_precentral_gyrus_ShapeIndex L_precentral_gyrus_Curvedness R_precentral_gyrus_AvgMeanCurvature R_precentral_gyrus_ComputeArea R_precentral_gyrus_Volume R_precentral_gyrus_ShapeIndex R_precentral_gyrus_Curvedness L_middle_orbitofrontal_gyrus_AvgMeanCurvature L_middle_orbitofrontal_gyrus_ComputeArea L_middle_orbitofrontal_gyrus_Volume L_middle_orbitofrontal_gyrus_ShapeIndex L_middle_orbitofrontal_gyrus_Curvedness R_middle_orbitofrontal_gyrus_AvgMeanCurvature R_middle_orbitofrontal_gyrus_ComputeArea R_middle_orbitofrontal_gyrus_Volume R_middle_orbitofrontal_gyrus_ShapeIndex R_middle_orbitofrontal_gyrus_Curvedness L_lateral_orbitofrontal_gyrus_AvgMeanCurvature L_lateral_orbitofrontal_gyrus_ComputeArea L_lateral_orbitofrontal_gyrus_Volume L_lateral_orbitofrontal_gyrus_ShapeIndex L_lateral_orbitofrontal_gyrus_Curvedness R_lateral_orbitofrontal_gyrus_AvgMeanCurvature R_lateral_orbitofrontal_gyrus_ComputeArea R_lateral_orbitofrontal_gyrus_Volume R_lateral_orbitofrontal_gyrus_ShapeIndex R_lateral_orbitofrontal_gyrus_Curvedness L_gyrus_rectus_AvgMeanCurvature L_gyrus_rectus_ComputeArea L_gyrus_rectus_Volume L_gyrus_rectus_ShapeIndex L_gyrus_rectus_Curvedness R_gyrus_rectus_AvgMeanCurvature R_gyrus_rectus_ComputeArea R_gyrus_rectus_Volume R_gyrus_rectus_ShapeIndex R_gyrus_rectus_Curvedness L_postcentral_gyrus_AvgMeanCurvature L_postcentral_gyrus_ComputeArea L_postcentral_gyrus_Volume L_postcentral_gyrus_ShapeIndex L_postcentral_gyrus_Curvedness R_postcentral_gyrus_AvgMeanCurvature R_postcentral_gyrus_ComputeArea R_postcentral_gyrus_Volume R_postcentral_gyrus_ShapeIndex R_postcentral_gyrus_Curvedness L_superior_parietal_gyrus_AvgMeanCurvature L_superior_parietal_gyrus_ComputeArea L_superior_parietal_gyrus_Volume L_superior_parietal_gyrus_ShapeIndex L_superior_parietal_gyrus_Curvedness R_superior_parietal_gyrus_AvgMeanCurvature R_superior_parietal_gyrus_ComputeArea R_superior_parietal_gyrus_Volume R_superior_parietal_gyrus_ShapeIndex R_superior_parietal_gyrus_Curvedness L_supramarginal_gyrus_AvgMeanCurvature L_supramarginal_gyrus_ComputeArea L_supramarginal_gyrus_Volume L_supramarginal_gyrus_ShapeIndex L_supramarginal_gyrus_Curvedness R_supramarginal_gyrus_AvgMeanCurvature R_supramarginal_gyrus_ComputeArea R_supramarginal_gyrus_Volume R_supramarginal_gyrus_ShapeIndex R_supramarginal_gyrus_Curvedness L_angular_gyrus_AvgMeanCurvature L_angular_gyrus_ComputeArea L_angular_gyrus_Volume L_angular_gyrus_ShapeIndex L_angular_gyrus_Curvedness R_angular_gyrus_AvgMeanCurvature R_angular_gyrus_ComputeArea R_angular_gyrus_Volume R_angular_gyrus_ShapeIndex R_angular_gyrus_Curvedness L_precuneus_AvgMeanCurvature L_precuneus_ComputeArea L_precuneus_Volume L_precuneus_ShapeIndex L_precuneus_Curvedness R_precuneus_AvgMeanCurvature R_precuneus_ComputeArea R_precuneus_Volume R_precuneus_ShapeIndex R_precuneus_Curvedness L_superior_occipital_gyrus_AvgMeanCurvature L_superior_occipital_gyrus_ComputeArea L_superior_occipital_gyrus_Volume L_superior_occipital_gyrus_ShapeIndex L_superior_occipital_gyrus_Curvedness R_superior_occipital_gyrus_AvgMeanCurvature R_superior_occipital_gyrus_ComputeArea R_superior_occipital_gyrus_Volume R_superior_occipital_gyrus_ShapeIndex R_superior_occipital_gyrus_Curvedness L_middle_occipital_gyrus_AvgMeanCurvature L_middle_occipital_gyrus_ComputeArea L_middle_occipital_gyrus_Volume L_middle_occipital_gyrus_ShapeIndex L_middle_occipital_gyrus_Curvedness R_middle_occipital_gyrus_AvgMeanCurvature R_middle_occipital_gyrus_ComputeArea R_middle_occipital_gyrus_Volume R_middle_occipital_gyrus_ShapeIndex R_middle_occipital_gyrus_Curvedness L_inferior_occipital_gyrus_AvgMeanCurvature L_inferior_occipital_gyrus_ComputeArea L_inferior_occipital_gyrus_Volume L_inferior_occipital_gyrus_ShapeIndex L_inferior_occipital_gyrus_Curvedness R_inferior_occipital_gyrus_AvgMeanCurvature R_inferior_occipital_gyrus_ComputeArea R_inferior_occipital_gyrus_Volume R_inferior_occipital_gyrus_ShapeIndex R_inferior_occipital_gyrus_Curvedness L_cuneus_AvgMeanCurvature L_cuneus_ComputeArea L_cuneus_Volume L_cuneus_ShapeIndex L_cuneus_Curvedness R_cuneus_AvgMeanCurvature R_cuneus_ComputeArea R_cuneus_Volume R_cuneus_ShapeIndex R_cuneus_Curvedness L_superior_temporal_gyrus_AvgMeanCurvature L_superior_temporal_gyrus_ComputeArea L_superior_temporal_gyrus_Volume L_superior_temporal_gyrus_ShapeIndex L_superior_temporal_gyrus_Curvedness R_superior_temporal_gyrus_AvgMeanCurvature R_superior_temporal_gyrus_ComputeArea R_superior_temporal_gyrus_Volume R_superior_temporal_gyrus_ShapeIndex R_superior_temporal_gyrus_Curvedness L_middle_temporal_gyrus_AvgMeanCurvature L_middle_temporal_gyrus_ComputeArea L_middle_temporal_gyrus_Volume L_middle_temporal_gyrus_ShapeIndex L_middle_temporal_gyrus_Curvedness R_middle_temporal_gyrus_AvgMeanCurvature R_middle_temporal_gyrus_ComputeArea R_middle_temporal_gyrus_Volume R_middle_temporal_gyrus_ShapeIndex R_middle_temporal_gyrus_Curvedness L_inferior_temporal_gyrus_AvgMeanCurvature L_inferior_temporal_gyrus_ComputeArea L_inferior_temporal_gyrus_Volume L_inferior_temporal_gyrus_ShapeIndex L_inferior_temporal_gyrus_Curvedness R_inferior_temporal_gyrus_AvgMeanCurvature R_inferior_temporal_gyrus_ComputeArea R_inferior_temporal_gyrus_Volume R_inferior_temporal_gyrus_ShapeIndex R_inferior_temporal_gyrus_Curvedness L_parahippocampal_gyrus_AvgMeanCurvature L_parahippocampal_gyrus_ComputeArea L_parahippocampal_gyrus_Volume L_parahippocampal_gyrus_ShapeIndex L_parahippocampal_gyrus_Curvedness R_parahippocampal_gyrus_AvgMeanCurvature R_parahippocampal_gyrus_ComputeArea R_parahippocampal_gyrus_Volume R_parahippocampal_gyrus_ShapeIndex R_parahippocampal_gyrus_Curvedness L_lingual_gyrus_AvgMeanCurvature L_lingual_gyrus_ComputeArea L_lingual_gyrus_Volume L_lingual_gyrus_ShapeIndex L_lingual_gyrus_Curvedness R_lingual_gyrus_AvgMeanCurvature R_lingual_gyrus_ComputeArea R_lingual_gyrus_Volume R_lingual_gyrus_ShapeIndex R_lingual_gyrus_Curvedness L_fusiform_gyrus_AvgMeanCurvature L_fusiform_gyrus_ComputeArea L_fusiform_gyrus_Volume L_fusiform_gyrus_ShapeIndex L_fusiform_gyrus_Curvedness R_fusiform_gyrus_AvgMeanCurvature R_fusiform_gyrus_ComputeArea R_fusiform_gyrus_Volume R_fusiform_gyrus_ShapeIndex R_fusiform_gyrus_Curvedness

**Demographics**: Sex Weight ResearchGroup VisitID Age

**Genetics**: chr12_rs34637584_GT chr12_rs34637584_DP chr12_rs34637584_GQ chr17_rs11868035_GT chr17_rs11868035_DP chr17_rs11868035_GQ chr17_rs11012_GT chr17_rs11012_DP chr17_rs11012_GQ chr17_rs393152_GT chr17_rs393152_DP chr17_rs393152_GQ chr17_rs12185268_GT chr17_rs12185268_DP chr17_rs12185268_GQ chr17_rs199533_GT chr17_rs199533_DP chr17_rs199533_GQ chr12_rs34637584_AD_1 chr12_rs34637584_AD_2 chr17_rs11868035_AD_1 chr17_rs11868035_AD_2 chr17_rs11012_AD_1 chr17_rs11012_AD_2 chr17_rs393152_AD_1 chr17_rs393152_AD_2 chr17_rs12185268_AD_1 chr17_rs12185268_AD_2 chr17_rs199533_AD_1 chr17_rs199533_AD_2 chr12_rs34637584_PL_1 chr12_rs34637584_PL_2 chr12_rs34637584_PL_3 chr17_rs11868035_PL_1 chr17_rs11868035_PL_2 chr17_rs11868035_PL_3 chr17_rs11012_PL_1 chr17_rs11012_PL_2 chr17_rs11012_PL_3 chr17_rs393152_PL_1 chr17_rs393152_PL_2 chr17_rs393152_PL_3 chr17_rs12185268_PL_1 chr17_rs12185268_PL_2 chr17_rs12185268_PL_3 chr17_rs199533_PL_1 chr17_rs199533_PL_2 chr17_rs199533_PL_3

**Uniform Parkinson’s Disease Rating Scale (UPDRS)**:

- **Top Level Variables**: UPDRS_Part_I_Summary_Score_Baseline UPDRS_Part_I_Summary_Score_Month_03 UPDRS_Part_I_Summary_Score_Month_06 UPDRS_Part_I_Summary_Score_Month_09 UPDRS_Part_I_Summary_Score_Month_12 UPDRS_Part_I_Summary_Score_Month_18 UPDRS_Part_I_Summary_Score_Month_24 UPDRS_Part_II_Patient_Questionnaire_Summary_Score_Baseline UPDRS_Part_II_Patient_Questionnaire_Summary_Score_Month_03 UPDRS_Part_II_Patient_Questionnaire_Summary_Score_Month_06 UPDRS_Part_II_Patient_Questionnaire_Summary_Score_Month_09 UPDRS_Part_II_Patient_Questionnaire_Summary_Score_Month_12 UPDRS_Part_II_Patient_Questionnaire_Summary_Score_Month_18 UPDRS_Part_II_Patient_Questionnaire_Summary_Score_Month_24 UPDRS_Part_III_Summary_Score_Baseline UPDRS_Part_III_Summary_Score_Month_03 UPDRS_Part_III_Summary_Score_Month_06 UPDRS_Part_III_Summary_Score_Month_09 UPDRS_Part_III_Summary_Score_Month_12 UPDRS_Part_III_Summary_Score_Month_18 UPDRS_Part_III_Summary_Score_Month_24 X_Assessment_Non.Motor_Epworth_Sleepiness_Scale_Summary_Score_Baseline X_Assessment_Non.Motor_Epworth_Sleepiness_Scale_Summary_Score_Month_06 X_Assessment_Non.Motor_Epworth_Sleepiness_Scale_Summary_Score_Month_12 X_Assessment_Non.Motor_Epworth_Sleepiness_Scale_Summary_Score_Month_24 X_Assessment_Non.Motor_Geriatric_Depression_Scale_GDS_Short_Summary_Score_Baseline X_Assessment_Non.Motor_Geriatric_Depression_Scale_GDS_Short_Summary_Score_Month_06 X_Assessment_Non.Motor_Geriatric_Depression_Scale_GDS_Short_Summary_Score_Month_12 X_Assessment_Non.Motor_Geriatric_Depression_Scale_GDS_Short_Summary_Score_Month_24
- **Middle-level variables**: (in addition to top-level) *HALLUCINATIONS_AND_PSYCHOSIS_* *_DEPRESSED_MOODS_* *Part_I_1.05_APATHY_* *_Part_I_1.06_FEATURES-

DOPAMINE_DYSREGULATION_SYNDROME_* UPDRS_Part_I_Summary_Score_Symptomatic_Therapy

*_Part_II_Patient_Questionnaire_2.04_EATING_TASKS_*

*_Part_II_Patient_Questionnaire_2.05_DRESSING_*

*_Part_II_Patient_Questionnaire_2.06_HYGIENE_*

*_Part_II_Patient_Questionnaire_2.07_HANDWRITING_*

*_Part_II_Patient_Questionnaire_2.08_DOING_HOBBIES_AND_OTHER_ACTIVITIES_*

*_Part_II_Patient_Questionnaire_2.09_TURNING_IN_BED_*

*_Part_III_3.01_Speech_* *_Part_III_3.03a_Rigidity_-_Neck_*

*_Part_III_3.09_Arising_from_chair_* *_Part_III_3.10_Gait_* *_Part_III_3.12_Postural_stability_*

*_Part_III_3.21_Hoehn_and_Yahr_Stage_NHY_*

*_Part_IV_4.2_Functional_impact_of_dyskinesias_* *_Part_IV_4.6_Painful_OFF-state_dystonia_*

*_Motor_Modified_Schwab_England_Activities_of_Daily_Living_Scale_*

*_Motor_Modified_Schwab_England_Activities_of_Daily_Living_Scale_Modified_Schwab_and_England_-_Overall_MSEADLG_* *_Overall_MSEADLG_*

*_Motor_Physical_Activity_Scale_for_the_Elderly_PASE_Household_Activity_Hours_worked_or_as_a_volunteer_WRKVLHR_*

*_Non-Motor_Semantic_Fluency_Total_Number_of_vegetable_VLTVEG_*

*_Non-Motor_State-Trait_Anxiety_Inventory_STAI_Question_01_STAIAD1_*

*_Non-Motor_State-Trait_Anxiety_Inventory_STAI_State_Subscore_*

- **Complete UPDRS**: a total of N=8191 variables.

* (asterisks denote varying times)

### **Table A**

**Table A** contains the complete classification results are based on different stratifications of the integrated PPMI dataset and represent *6 alternative data mining methods* including: **adaboost [**[**56**](#_ENREF_56)**], svm [**[**57**](#_ENREF_57)**], naïve Bayes [**[**58**](#_ENREF_58)**], decision tree [**[**59**](#_ENREF_59)**], knn [**[**60**](#_ENREF_60)**]** and **k-means [**[**61**](#_ENREF_61)**]**. Two *types of data strata* represent unbalanced datasets extracted from the original (unbalanced) and statistically re-balanced data (no balance label). Further *two groups*, PD alone or PD+SWEED, are compared to controls. Thus, the total number of results in the table below includes $N=24$ rows, corresponding to 6(methods) x 2(types) x 2(groups). The cell values represent the averages of the corresponding 5-fold cross-validation measures.

| Table S1.A: Machine learning based classification results representing 5-fold cross-validation average measures. | | | | | | | | | | | | |
| --- | --- | --- | --- | --- | --- | --- | --- | --- | --- | --- | --- | --- |
| classifier | **Cohorts** | **Balance** | **FP** | **TP** | **TN** | **FN** | **Accuracy** | **Sensitivity** | **Specificity** | **PPV** | **NPV** | **LOR** |
| adaboost | PD vs. HC | balanced | 0.2 | 72.2 | 98.2 | 1.6 | 0.98954704 | 0.97831978 | 0.99796748 | 0.99723757 | 0.98396794 | 10.0058805 |
| detree | PD vs. HC | balanced | 191.2 | 134 | 5.6 | 13.6 | 0.40534262 | 0.90785908 | 0.02845528 | 0.41205412 | 0.29166667 | -1.2427834 |
| nb | PD vs. HC | balanced | 45 | 63.4 | 53.4 | 10.4 | 0.67828107 | 0.85907859 | 0.54268293 | 0.58487085 | 0.8369906 | 1.97880631 |
| svm | PD vs. HC | balanced | 2 | 69.4 | 96.4 | 4.4 | 0.96283391 | 0.9403794 | 0.9796748 | 0.9719888 | 0.95634921 | 6.63364135 |
| knn | PD vs. HC | balanced | 17.8 | 55.2 | 80.6 | 18.6 | 0.78861789 | 0.74796748 | 0.81910569 | 0.75616438 | 0.8125 | 2.59810156 |
| kmeans | PD vs. HC | balanced | 163.6 | 126 | 231.6 | 167.6 | 0.51916376 | 0.42915531 | 0.58603239 | 0.43508287 | 0.58016032 | 0.06228904 |
| adaboost / wo clinical data | PD vs. HC | balanced | 2.8 | 58 | 95.6 | 15.8 | 0.89198606 | 0.78590786 | 0.97154472 | 0.95394737 | 0.85816876 | 4.83098647 |
| adaboost | PD vs. HC | unbalanced | 0.4 | 22.4 | 52.2 | 2.2 | 0.96632124 | 0.91056911 | 0.99239544 | 0.98245614 | 0.95955882 | 7.19197683 |
| detree | PD vs. HC | unbalanced | 93.4 | 44 | 11.8 | 5.2 | 0.36139896 | 0.89430894 | 0.1121673 | 0.3202329 | 0.69411765 | 0.06673919 |
| nb | PD vs. HC | unbalanced | 9.4 | 8.6 | 43.2 | 16 | 0.67098446 | 0.3495935 | 0.82129278 | 0.47777778 | 0.72972973 | 0.90430429 |
| svm | PD vs. HC | unbalanced | 1 | 7 | 51.6 | 17.6 | 0.75906736 | 0.28455285 | 0.98098859 | 0.875 | 0.74566474 | 3.02153292 |
| knn | PD vs. HC | unbalanced | 15.2 | 7.6 | 37.4 | 17 | 0.58290155 | 0.30894309 | 0.71102662 | 0.33333333 | 0.6875 | 0.09531018 |
| kmeans | PD vs. HC | unbalanced | 87.6 | 38.4 | 124.4 | 58.4 | 0.52720207 | 0.39669421 | 0.58679245 | 0.3047619 | 0.68052516 | -0.0685372 |
| adaboost / wo clinical data | PD vs. HC | unbalanced | 4 | 4 | 48.6 | 20.6 | 0.68134715 | 0.16260163 | 0.92395437 | 0.5 | 0.70231214 | 0.85833246 |
| adaboost | PD+SWEDD vs. HC | balanced | 1.2 | 72 | 97.2 | 1.8 | 0.9825784 | 0.97560976 | 0.98780488 | 0.98360656 | 0.98181818 | 8.08332861 |
| detree | PD+SWEDD vs. HC | balanced | 195.8 | 146.4 | 1 | 1.2 | 0.42799071 | 0.99186992 | 0.0050813 | 0.42781999 | 0.45454545 | -0.4730727 |
| nb | PD+SWEDD vs. HC | balanced | 43.6 | 64 | 54.8 | 9.8 | 0.68989547 | 0.86720867 | 0.55691057 | 0.59479554 | 0.84829721 | 2.10513374 |
| svm | PD+SWEDD vs. HC | balanced | 2.8 | 67.2 | 95.6 | 6.6 | 0.94541231 | 0.91056911 | 0.97154472 | 0.96 | 0.93542074 | 5.851157 |
| knn | PD+SWEDD vs. HC | balanced | 20.4 | 56.2 | 78 | 17.6 | 0.77932636 | 0.76151762 | 0.79268293 | 0.73368146 | 0.81589958 | 2.50219178 |
| kmeans | PD+SWEDD vs. HC | balanced | 165.4 | 124.2 | 229.8 | 169.4 | 0.51393728 | 0.42302452 | 0.58147773 | 0.4288674 | 0.5756513 | 0.01847297 |
| adaboost / wo clinical data | PD+SWEDD vs. HC | balanced | 4.6 | 55.8 | 93.8 | 18 | 0.86875726 | 0.75609756 | 0.95325203 | 0.92384106 | 0.83899821 | 4.14651066 |
| adaboost | PD+SWEDD vs. HC | unbalanced | 0.8 | 22.4 | 59.2 | 2.2 | 0.96453901 | 0.91056911 | 0.98666667 | 0.96551724 | 0.96416938 | 6.62466869 |
| detree | PD+SWEDD vs. HC | unbalanced | 120 | 49.2 | 0 | 0 | 0.29078014 | 1 | 0 | 0.29078014 | NA | NA |
| nb | PD+SWEDD vs. HC | unbalanced | 12.4 | 10.6 | 47.6 | 14 | 0.68794326 | 0.43089431 | 0.79333333 | 0.46086957 | 0.77272727 | 1.06693296 |
| svm | PD+SWEDD vs. HC | unbalanced | 0.8 | 3.2 | 59.2 | 21.4 | 0.73758865 | 0.1300813 | 0.98666667 | 0.8 | 0.73449132 | 2.40382498 |
| knn | PD+SWEDD vs. HC | unbalanced | 17.2 | 7.2 | 42.8 | 17.4 | 0.59101655 | 0.29268293 | 0.71333333 | 0.29508197 | 0.71096346 | 0.02923954 |
| kmeans | PD+SWEDD vs. HC | unbalanced | 114.2 | 44.6 | 127 | 52.6 | 0.5070922 | 0.45884774 | 0.526534 | 0.28085642 | 0.70712695 | -0.0587465 |
| adaboost / wo clinical data | PD+SWEDD vs. HC | unbalanced | 4.2 | 2 | 55.8 | 22.6 | 0.68321513 | 0.08130081 | 0.93 | 0.32258065 | 0.71173469 | 0.16188662 |

### **Fig A**


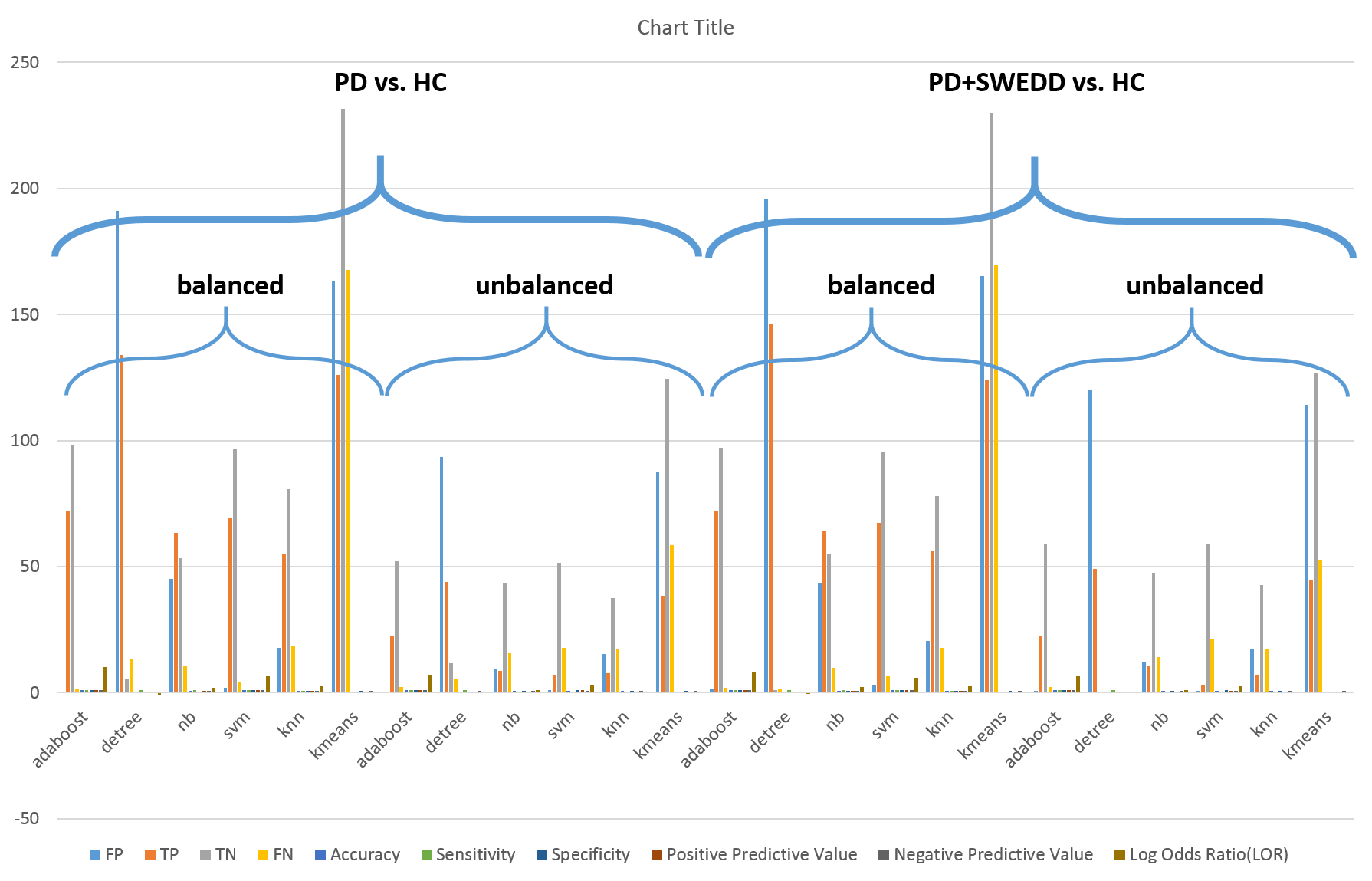


**Figure A**: Graphical depiction of the numerical results included in **Table A**.

### Data B

Classifier information of AdaBoost with UPDRS included: For the UPDRS-based analyses, only UPDRS “Part I”, “Part II”, and “Part III” data elements were included.

**AdaBoost Classifier model (based on RWeka), balanced design, PD+SWEDD vs. HC**

| Data | Cohorts | Balance | FP | TP | TN | FN | Accuracy | Sensitivity | Specificity | PPV | NPV | LOR |
| --- | --- | --- | --- | --- | --- | --- | --- | --- | --- | --- | --- | --- |
| With UPDRS | HC vs. PD | unbalanced | 0.4 | 22.4 | 52.2 | 2.2 | 0.96632124 | 0.91056911 | 0.99239544 | 0.98245614 | 0.95955882 | 7.19 |
|  | HC vs. PD+SWEDD |  | 0.8 | 22.4 | 59.2 | 2.2 | 0.96453901 | 0.91056911 | 0.98666667 | 0.96551724 | 0.96416938 | 6.62 |
|  | HC vs. PD | balanced | 0.2 | 72.2 | 98.2 | 1.6 | 0.98954704 | 0.97831978 | 0.99796748 | 0.99723757 | 0.98396794 | 10.0 |
|  | HC vs. PD+SWEDD |  | 1.2 | 72 | 97.2 | 1.8 | 0.9825784 | 0.97560976 | 0.98780488 | 0.98360656 | 0.98181818 | 8.09 |
| Without UPDRS | HC vs. PD | unbalanced | 4 | 4 | 48.6 | 20.6 | 0.68134715 | 0.16260163 | 0.92395437 | 0.5 | 0.70231214 | 0.86 |
|  | HC vs. PD+SWEDD |  | 4.2 | 2 | 55.8 | 22.6 | 0.68321513 | 0.08130081 | 0.93 | 0.32258065 | 0.71173469 | 0.16 |
|  | HC vs. PD | balanced | 2.8 | 58 | 95.6 | 15.8 | 0.89198606 | 0.78590786 | 0.97154472 | 0.95394737 | 0.85816876 | 4.83 |
|  | HC vs. PD+SWEDD |  | 4.6 | 55.8 | 93.8 | 18 | 0.86875726 | 0.75609756 | 0.95325203 | 0.92384106 | 0.83899821 | 4.15 |

Classifier (AdaBoost with UPDRS data)

AdaBoostM1: Base classifiers and their weights:

Decision Stump

Classifications

UPDRS_Part_III_Summary_Score_Baseline <= 8.94208837579936 : 1

UPDRS_Part_III_Summary_Score_Baseline > 8.94208837579936 : 0

UPDRS_Part_III_Summary_Score_Baseline is missing : 0

Class distributions

UPDRS_Part_III_Summary_Score_Baseline <= 8.94208837579936

0 1

0.12437810945273632 0.8756218905472637

UPDRS_Part_III_Summary_Score_Baseline > 8.94208837579936

0 1

0.9629629629629629 0.037037037037037035

UPDRS_Part_III_Summary_Score_Baseline is missing

0 1

0.5714285714285714 0.42857142857142855

Weight: 2.47

Decision Stump

Classifications

UPDRS_Part_II_Patient_Questionnaire_Summary_Score_Baseline <= 0.9839244195027275 : 1

UPDRS_Part_II_Patient_Questionnaire_Summary_Score_Baseline > 0.9839244195027275 : 0

UPDRS_Part_II_Patient_Questionnaire_Summary_Score_Baseline is missing : 0

Class distributions

UPDRS_Part_II_Patient_Questionnaire_Summary_Score_Baseline <= 0.9839244195027275

0 1

0.058217354931933876 0.9417826450680661

UPDRS_Part_II_Patient_Questionnaire_Summary_Score_Baseline > 0.9839244195027275

0 1

0.8462508427176707 0.1537491572823292

UPDRS_Part_II_Patient_Questionnaire_Summary_Score_Baseline is missing

0 1

0.6514718598443531 0.348528140155647

Weight: 1.9

Decision Stump

Classifications

UPDRS_Part_II_Patient_Questionnaire_Summary_Score_Baseline <= 2.97110035037622 : 1

UPDRS_Part_II_Patient_Questionnaire_Summary_Score_Baseline > 2.97110035037622 : 0

UPDRS_Part_II_Patient_Questionnaire_Summary_Score_Baseline is missing : 1

Class distributions

UPDRS_Part_II_Patient_Questionnaire_Summary_Score_Baseline <= 2.97110035037622

0 1

0.2601928224059411 0.7398071775940589

UPDRS_Part_II_Patient_Questionnaire_Summary_Score_Baseline > 2.97110035037622

0 1

0.938912385605746 0.061087614394254065

UPDRS_Part_II_Patient_Questionnaire_Summary_Score_Baseline is missing

0 1

0.42148337586727463 0.5785166241327253

Weight: 1.31

Decision Stump

Classifications

Age <= 72.0507 : 0

Age > 72.0507 : 1

Age is missing : 0

Class distributions

Age <= 72.0507

0 1

0.7640616067131697 0.2359383932868304

Age > 72.0507

0 1

0.08386773377385388 0.9161322662261461

Age is missing

0 1

0.6076364602796261 0.39236353972037386

Weight: 1.38

Decision Stump

Classifications

UPDRS_Part_III_Summary_Score_Baseline <= 1.999596913461575 : 1

UPDRS_Part_III_Summary_Score_Baseline > 1.999596913461575 : 0

UPDRS_Part_III_Summary_Score_Baseline is missing : 1

Class distributions

UPDRS_Part_III_Summary_Score_Baseline <= 1.999596913461575

0 1

0.0 1.0

UPDRS_Part_III_Summary_Score_Baseline > 1.999596913461575

0 1

0.5272557461241035 0.4727442538758964

UPDRS_Part_III_Summary_Score_Baseline is missing

0 1

0.41614914448140444 0.5838508555185956

Weight: 0.52

Decision Stump

Classifications

cerebellum_AvgMeanCurvature <= -0.012134046709148302 : 1

cerebellum_AvgMeanCurvature > -0.012134046709148302 : 1

cerebellum_AvgMeanCurvature is missing : 1

Class distributions

cerebellum_AvgMeanCurvature <= -0.012134046709148302

0 1

0.008874493051233013 0.9911255069487669

cerebellum_AvgMeanCurvature > -0.012134046709148302

0 1

0.4325804419851906 0.5674195580148095

cerebellum_AvgMeanCurvature is missing

0 1

0.3319233607096255 0.6680766392903744

Weight: 0.7

Decision Stump

Classifications

cerebellum_AvgMeanCurvature <= -0.012134046709148302 : 1

cerebellum_AvgMeanCurvature > -0.012134046709148302 : 0

cerebellum_AvgMeanCurvature is missing : 1

Class distributions

cerebellum_AvgMeanCurvature <= -0.012134046709148302

0 1

0.017702972610309446 0.9822970273896906

cerebellum_AvgMeanCurvature > -0.012134046709148302

0 1

0.6054361443698247 0.39456385563017515

cerebellum_AvgMeanCurvature is missing

0 1

0.4999999999999979 0.5000000000000021

Weight: 0.72

Decision Stump

Classifications

UPDRS_Part_III_Summary_Score_Baseline <= 1.999596913461575 : 1

UPDRS_Part_III_Summary_Score_Baseline > 1.999596913461575 : 1

UPDRS_Part_III_Summary_Score_Baseline is missing : 1

Class distributions

UPDRS_Part_III_Summary_Score_Baseline <= 1.999596913461575

0 1

0.0 1.0

UPDRS_Part_III_Summary_Score_Baseline > 1.999596913461575

0 1

0.4594906600571509 0.5405093399428492

UPDRS_Part_III_Summary_Score_Baseline is missing

0 1

0.37394465345305383 0.6260553465469462

Weight: 0.52

Decision Stump

Classifications

UPDRS_Part_III_Summary_Score_Baseline <= 1.999596913461575 : 1

UPDRS_Part_III_Summary_Score_Baseline > 1.999596913461575 : 0

UPDRS_Part_III_Summary_Score_Baseline is missing : 0

Class distributions

UPDRS_Part_III_Summary_Score_Baseline <= 1.999596913461575

0 1

0.0 1.0

UPDRS_Part_III_Summary_Score_Baseline > 1.999596913461575

0 1

0.5873297762793167 0.41267022372068324

UPDRS_Part_III_Summary_Score_Baseline is missing

0 1

0.5000000000000012 0.4999999999999987

Weight: 0.61

Decision Stump

Classifications

cerebellum_ComputeArea <= 18739.6455 : 1

cerebellum_ComputeArea > 18739.6455 : 0

cerebellum_ComputeArea is missing : 1

Class distributions

cerebellum_ComputeArea <= 18739.6455

0 1

0.2874211506643006 0.7125788493356994

cerebellum_ComputeArea > 18739.6455

0 1

0.8153484011802252 0.1846515988197747

cerebellum_ComputeArea is missing

0 1

0.3853923894019641 0.6146076105980359

Weight: 1.0

Number of performed Iterations: 10

Time taken to build model: 0.63 seconds

=== Stratified cross-validation ===

=== Summary ===

Correctly Classified Instances 828 96.1672 %

Incorrectly Classified Instances 33 3.8328 %

Kappa statistic 0.9218

Mean absolute error 0.0556

Root mean squared error 0.1693

Relative absolute error 11.3517 %

Root relative squared error 34.2183 %

Total Number of Instances 861

=== Detailed Accuracy By Class ===

TP Rate FP Rate Precision Recall F-Measure ROC Area Class

0.965 0.043 0.967 0.965 0.966 0.994 0

0.957 0.035 0.954 0.957 0.955 0.994 1

Weighted Avg. 0.962 0.04 0.962 0.962 0.962 0.994

=== Confusion Matrix ===

a b <-- classified as

475 17 | a = 0

16 353 | b = 1

### Data C

**Classifier information of AdaBoost without UPDRS variables: AdaBoost Classifier model (based on RWeka), balanced design, PD+SWEDD vs. HC**

These exclude UPDRS “Part I”, “Part II”, “Part III”, “Part IV” data elements, but include others like “*X_Assessment_Non.Motor_Epworth_Sleepiness_Scale_Summary_Score*”.

AdaBoostM1: Base classifiers and their weights:

Decision Stump

Classifications

COGSTATE <= 2.5 : 0

COGSTATE > 2.5 : 0

COGSTATE is missing : 0

Class distributions

COGSTATE <= 2.5

0 1

0.9764705882352941 0.023529411764705882

COGSTATE > 2.5

0 1

0.5270618556701031 0.4729381443298969

COGSTATE is missing

0 1

0.5714285714285714 0.42857142857142855

Weight: 0.29

Decision Stump

Classifications

COGSTATE <= 2.5 : 0

COGSTATE > 2.5 : 1

COGSTATE is missing : 1

Class distributions

COGSTATE <= 2.5

0 1

0.9688715953307394 0.031128404669260715

COGSTATE > 2.5

0 1

0.4552875695732831 0.544712430426717

COGSTATE is missing

0 1

0.4999999999999992 0.5000000000000008

Weight: 0.33

Decision Stump

Classifications

L_insular_cortex_Curvedness <= 0.1052903013537405 : 0

L_insular_cortex_Curvedness > 0.1052903013537405 : 1

L_insular_cortex_Curvedness is missing : 0

Class distributions

L_insular_cortex_Curvedness <= 0.1052903013537405

0 1

0.7858702542790722 0.21412974572092772

L_insular_cortex_Curvedness > 0.1052903013537405

0 1

0.4743835584387291 0.525616441561271

L_insular_cortex_Curvedness is missing

0 1

0.5692713257989254 0.4307286742010747

Weight: 0.43

Decision Stump

Classifications

EDUCYRS <= 12.06716133700685 : 0

EDUCYRS > 12.06716133700685 : 0

EDUCYRS is missing : 0

Class distributions

EDUCYRS <= 12.06716133700685

0 1

0.9429536761023022 0.057046323897697856

EDUCYRS > 12.06716133700685

0 1

0.5710018615094445 0.4289981384905555

EDUCYRS is missing

0 1

0.6153354869700528 0.38466451302994714

Weight: 0.47

Decision Stump

Classifications

EDUCYRS <= 12.06716133700685 : 0

EDUCYRS > 12.06716133700685 : 1

EDUCYRS is missing : 0

Class distributions

EDUCYRS <= 12.06716133700685

0 1

0.9117633009969731 0.08823669900302691

EDUCYRS > 12.06716133700685

0 1

0.4541649968940542 0.5458350031059458

EDUCYRS is missing

0 1

0.5000000000000089 0.49999999999999106

Weight: 0.33

Decision Stump

Classifications

L_inferior_frontal_gyrus_AvgMeanCurvature <= 0.08996533949999999 : 1

L_inferior_frontal_gyrus_AvgMeanCurvature > 0.08996533949999999 : 0

L_inferior_frontal_gyrus_AvgMeanCurvature is missing : 0

Class distributions

L_inferior_frontal_gyrus_AvgMeanCurvature <= 0.08996533949999999

0 1

0.38867408854175767 0.6113259114582423

L_inferior_frontal_gyrus_AvgMeanCurvature > 0.08996533949999999

0 1

0.6680630772600715 0.33193692273992836

L_inferior_frontal_gyrus_AvgMeanCurvature is missing

0 1

0.5678088108719184 0.43219118912808163

Weight: 0.61

Decision Stump

Classifications

L_middle_occipital_gyrus_AvgMeanCurvature <= 0.0854281039628689 : 0

L_middle_occipital_gyrus_AvgMeanCurvature > 0.0854281039628689 : 1

L_middle_occipital_gyrus_AvgMeanCurvature is missing : 0

Class distributions

L_middle_occipital_gyrus_AvgMeanCurvature <= 0.0854281039628689

0 1

0.7757771320302077 0.22422286796979227

L_middle_occipital_gyrus_AvgMeanCurvature > 0.0854281039628689

0 1

0.45659388545463087 0.5434061145453691

L_middle_occipital_gyrus_AvgMeanCurvature is missing

0 1

0.5286033786406487 0.4713966213593513

Weight: 0.39

Decision Stump

Classifications

L_precentral_gyrus_ShapeIndex <= 0.34140097999999997 : 1

L_precentral_gyrus_ShapeIndex > 0.34140097999999997 : 0

L_precentral_gyrus_ShapeIndex is missing : 0

Class distributions

L_precentral_gyrus_ShapeIndex <= 0.34140097999999997

0 1

0.4755105726885129 0.5244894273114871

L_precentral_gyrus_ShapeIndex > 0.34140097999999997

0 1

0.7340602951051085 0.26593970489489144

L_precentral_gyrus_ShapeIndex is missing

0 1

0.5842902914642467 0.4157097085357533

Weight: 0.46

Decision Stump

Classifications

COGSTATE <= 2.5 : 0

COGSTATE > 2.5 : 0

COGSTATE is missing : 0

Class distributions

COGSTATE <= 2.5

0 1

0.9731504655653223 0.02684953443467772

COGSTATE > 2.5

0 1

0.574322414695629 0.425677585304371

COGSTATE is missing

0 1

0.6076154414191902 0.3923845585808098

Weight: 0.44

Decision Stump

Classifications

COGSTATE <= 2.5 : 0

COGSTATE > 2.5 : 1

COGSTATE is missing : 0

Class distributions

COGSTATE <= 2.5

0 1

0.9590263911579866 0.04097360884201338

COGSTATE > 2.5

0 1

0.46560662756137233 0.5343933724386276

COGSTATE is missing

0 1

0.5000000000000002 0.4999999999999999

Weight: 0.26

Number of performed Iterations: 10

Time taken to build model: 0.65 seconds

=== Stratified cross-validation ===

=== Summary ===

Correctly Classified Instances 611 70.964 %

Incorrectly Classified Instances 250 29.036 %

Kappa statistic 0.4048

Mean absolute error 0.3838

Root mean squared error 0.4303

Relative absolute error 78.3447 %

Root relative squared error 86.96 %

Total Number of Instances 861

=== Detailed Accuracy By Class ===

TP Rate FP Rate Precision Recall F-Measure ROC Area Class

0.758 0.355 0.74 0.758 0.749 0.781 0

0.645 0.242 0.667 0.645 0.656 0.781 1

Weighted Avg. 0.71 0.307 0.709 0.71 0.709 0.781

=== Confusion Matrix ===

a b <-- classified as

373 119 | a = 0

131 238 | b = 1

### Data D

The classifier (synthesis) sub-element of RWeka model is a Java object which is not easy to use as a separate module from the learning model (analysis) component, however WEKA’s graphical user interface does allow independent invocation of the analysis and synthesis components of the AdaBoost classifier. In our experiments, we used a default number of iteration = 10 (10 weights are created).

The **2^3^=8 Study Designs** were used based on the following stratifications:

- **Comparison**: Controls vs PD (alone) or Controls vs (PD+SWEDD)
- **Data Size**: Unbalanced (default) vs. Balanced (statistically re-balanced sample-sizes)
- **Variables**: with-UPDRS scores (including Parts I, II, and III variables) or without-UPDRS scores

**Dataset Labeling (Comparison * Data Size):**

- **1=PDvsControl unbalanced,**
- **2=PD and SWEDDvsControl unbalanced,**
- **3=PDvsControl balanced,**
- **4=PD and SWEDDvsControl balanced**

From the model weight information, besides UPDRS scores, there are **3 imaging indexes** accounting for the prediction of the final outcome consistently (across study-designs). The consistency comes from the dataset 2 and dataset 4, respectively. These are the unbalanced and balanced datasets of PD+SWEDD vs Control stratification. These imaging biomarkers are:

- ***R_middle_orbitofrontal_gyrus_AvgMeanCurvature***
- ***L_supramarginal_gyrus_ShapeIndex,***
- ***L_superior_occipital_gyrus_AvgMeanCurvature.***

In addition, the *“****Age”*** shows great consistency of the 4 datasets.

We tried several alternative classifiers with AdaBoost uniformly outperforming most others.

AdaBoost Model

The following graphs (Varplots) represent the variable importance by their “scores” based on different study-designs. The varplots based on the raw (imbalanced) dataset show that the importance of the data elements is highly non-linear, whereas the plots of the corresponding importance scores using the rebalanced dataset are more linear.

### **Fig B**

Using the prediction model **with the UPDRS scores, balanced, PD+SWEDD vs. Control**

### **Fig C**

Using the prediction model **without the UPDRS scores, balanced, PD+SWEDD vs. Control**

### **Fig D**

Using the prediction model **with the UPDRS scores, balanced, PD vs. Control**

### **Fig E**

Using the prediction model **without the UPDRS scores, balanced, PD vs. Control**

### **Fig F**

Using the prediction model **with the UPDRS scores, unbalanced, PD+SWEDD vs. Control**

### **Fig G**

Using the prediction model **without the UPDRS scores, unbalanced, PD+SWEDD vs. Control**

### **Fig H**

Using the prediction model **with the UPDRS scores, unbalanced, PD vs. Control**

### **Fig I**

Using the prediction model **without the UPDRS scores, unbalanced, PD vs. Control**

### Table B

**Table B** includes all data elements that frequently showed up as important (top-30) predictors of subject diagnosis (see text).

| Variable Names | PD vs HC | PD+SWEDD vs HC | Re-balanced | Im-balanced | With Clinical data | Without Clinical data |
| --- | --- | --- | --- | --- | --- | --- |
| Age | 0 | 2 | 1 | 1 | 2 | 0 |
| brainstem_AvgMeanCurvature | 1 | 1 | 0 | 2 | 1 | 1 |
| brainstem_ComputeArea | 0 | 2 | 0 | 2 | 1 | 1 |
| brainstem_Volume | 0 | 1 | 0 | 1 | 0 | 1 |
| cerebellum_AvgMeanCurvature | 1 | 1 | 2 | 0 | 2 | 0 |
| cerebellum_ComputeArea | 0 | 1 | 0 | 1 | 1 | 0 |
| cerebellum_Curvedness | 1 | 2 | 3 | 0 | 2 | 1 |
| cerebellum_Volume | 2 | 0 | 1 | 1 | 2 | 0 |
| EDUCYRS | 1 | 0 | 0 | 1 | 1 | 0 |
| L_angular_gyrus_Volume | 0 | 1 | 0 | 1 | 0 | 1 |
| L_caudate_AvgMeanCurvature | 1 | 0 | 0 | 1 | 1 | 0 |
| L_caudate_ComputeArea | 1 | 1 | 2 | 0 | 1 | 1 |
| L_caudate_Curvedness | 1 | 0 | 1 | 0 | 1 | 0 |
| L_caudate_Volume | 0 | 1 | 1 | 0 | 0 | 1 |
| L_cingulate_gyrus_ComputeArea | 0 | 1 | 1 | 0 | 0 | 1 |
| L_cingulate_gyrus_Volume | 0 | 1 | 1 | 0 | 0 | 1 |
| L_cuneus_ComputeArea | 0 | 1 | 0 | 1 | 0 | 1 |
| L_cuneus_Curvedness | 0 | 1 | 0 | 1 | 0 | 1 |
| L_fusiform_gyrus_ComputeArea | 1 | 0 | 0 | 1 | 0 | 1 |
| L_fusiform_gyrus_ShapeIndex | 2 | 0 | 1 | 1 | 2 | 0 |
| L_gyrus_rectus_AvgMeanCurvature | 0 | 2 | 1 | 1 | 2 | 0 |
| L_gyrus_rectus_ShapeIndex | 0 | 2 | 1 | 1 | 2 | 0 |
| L_hippocampus_AvgMeanCurvature | 1 | 1 | 1 | 1 | 0 | 2 |
| L_hippocampus_ComputeArea | 3 | 3 | 3 | 3 | 4 | 2 |
| L_hippocampus_Curvedness | 2 | 1 | 2 | 1 | 2 | 1 |
| L_hippocampus_ShapeIndex | 1 | 2 | 2 | 1 | 3 | 0 |
| L_hippocampus_Volume | 3 | 3 | 3 | 3 | 4 | 2 |
| L_inferior_frontal_gyrus_AvgMeanCurvature | 0 | 1 | 1 | 0 | 1 | 0 |
| L_inferior_frontal_gyrus_ComputeArea | 1 | 1 | 2 | 0 | 0 | 2 |
| L_inferior_frontal_gyrus_Curvedness | 2 | 0 | 1 | 1 | 1 | 1 |
| L_inferior_frontal_gyrus_ShapeIndex | 1 | 0 | 1 | 0 | 1 | 0 |
| L_inferior_frontal_gyrus_Volume | 2 | 3 | 3 | 2 | 1 | 4 |
| L_inferior_temporal_gyrus_AvgMeanCurvature | 1 | 1 | 2 | 0 | 2 | 0 |
| L_inferior_temporal_gyrus_ShapeIndex | 1 | 1 | 0 | 2 | 2 | 0 |
| L_insular_cortex_ComputeArea | 1 | 2 | 1 | 2 | 0 | 3 |
| L_insular_cortex_Curvedness | 0 | 2 | 0 | 2 | 1 | 1 |
| L_insular_cortex_Volume | 0 | 1 | 1 | 0 | 0 | 1 |
| L_lateral_orbitofrontal_gyrus_ComputeArea | 1 | 1 | 1 | 1 | 0 | 2 |
| L_lateral_orbitofrontal_gyrus_ShapeIndex | 1 | 1 | 0 | 2 | 2 | 0 |
| L_lateral_orbitofrontal_gyrus_Volume | 1 | 2 | 1 | 2 | 3 | 0 |
| L_lingual_gyrus_AvgMeanCurvature | 2 | 0 | 1 | 1 | 0 | 2 |
| L_lingual_gyrus_ComputeArea | 1 | 0 | 0 | 1 | 0 | 1 |
| L_middle_frontal_gyrus_AvgMeanCurvature | 0 | 1 | 1 | 0 | 1 | 0 |
| L_middle_frontal_gyrus_Curvedness | 1 | 1 | 1 | 1 | 0 | 2 |
| L_middle_occipital_gyrus_ComputeArea | 1 | 1 | 2 | 0 | 0 | 2 |
| L_middle_occipital_gyrus_Volume | 1 | 2 | 2 | 1 | 0 | 3 |
| L_middle_orbitofrontal_gyrus_ComputeArea | 1 | 1 | 0 | 2 | 0 | 2 |
| L_parahippocampal_gyrus_AvgMeanCurvature | 1 | 0 | 1 | 0 | 1 | 0 |
| L_parahippocampal_gyrus_ComputeArea | 2 | 2 | 2 | 2 | 4 | 0 |
| L_parahippocampal_gyrus_Curvedness | 1 | 2 | 1 | 2 | 3 | 0 |
| L_parahippocampal_gyrus_ShapeIndex | 1 | 0 | 1 | 0 | 1 | 0 |
| L_parahippocampal_gyrus_Volume | 3 | 3 | 3 | 3 | 3 | 3 |
| L_precentral_gyrus_AvgMeanCurvature | 1 | 2 | 1 | 2 | 2 | 1 |
| L_precentral_gyrus_ShapeIndex | 0 | 1 | 1 | 0 | 1 | 0 |
| L_precentral_gyrus_Volume | 0 | 1 | 0 | 1 | 0 | 1 |
| L_precuneus_AvgMeanCurvature | 1 | 0 | 1 | 0 | 0 | 1 |
| L_precuneus_Curvedness | 0 | 2 | 1 | 1 | 0 | 2 |
| L_putamen_AvgMeanCurvature | 2 | 1 | 3 | 0 | 1 | 2 |
| L_putamen_ComputeArea | 1 | 2 | 2 | 1 | 0 | 3 |
| L_putamen_ShapeIndex | 1 | 0 | 0 | 1 | 1 | 0 |
| L_putamen_Volume | 1 | 2 | 2 | 1 | 0 | 3 |
| L_superior_frontal_gyrus_AvgMeanCurvature | 1 | 0 | 1 | 0 | 0 | 1 |
| L_superior_occipital_gyrus_ComputeArea | 0 | 1 | 0 | 1 | 0 | 1 |
| L_superior_occipital_gyrus_ShapeIndex | 2 | 1 | 2 | 1 | 3 | 0 |
| L_superior_parietal_gyrus_AvgMeanCurvature | 1 | 0 | 1 | 0 | 0 | 1 |
| L_superior_parietal_gyrus_ComputeArea | 1 | 0 | 0 | 1 | 1 | 0 |
| L_superior_parietal_gyrus_Volume | 1 | 0 | 0 | 1 | 1 | 0 |
| L_supramarginal_gyrus_ComputeArea | 0 | 1 | 1 | 0 | 1 | 0 |
| L_supramarginal_gyrus_Volume | 1 | 1 | 1 | 1 | 1 | 1 |
| R_angular_gyrus_ComputeArea | 2 | 0 | 1 | 1 | 1 | 1 |
| R_angular_gyrus_Curvedness | 1 | 0 | 0 | 1 | 1 | 0 |
| R_angular_gyrus_ShapeIndex | 1 | 0 | 1 | 0 | 1 | 0 |
| R_angular_gyrus_Volume | 0 | 1 | 0 | 1 | 0 | 1 |
| R_caudate_AvgMeanCurvature | 1 | 0 | 0 | 1 | 0 | 1 |
| R_caudate_ComputeArea | 1 | 0 | 0 | 1 | 0 | 1 |
| R_caudate_Curvedness | 1 | 0 | 0 | 1 | 0 | 1 |
| R_caudate_Volume | 1 | 0 | 0 | 1 | 0 | 1 |
| R_cingulate_gyrus_ComputeArea | 1 | 0 | 1 | 0 | 0 | 1 |
| R_cingulate_gyrus_Volume | 2 | 0 | 1 | 1 | 1 | 1 |
| R_cuneus_ComputeArea | 0 | 1 | 0 | 1 | 0 | 1 |
| R_fusiform_gyrus_ComputeArea | 1 | 0 | 1 | 0 | 0 | 1 |
| R_fusiform_gyrus_Volume | 0 | 1 | 0 | 1 | 0 | 1 |
| R_gyrus_rectus_ComputeArea | 1 | 1 | 0 | 2 | 0 | 2 |
| R_gyrus_rectus_Curvedness | 0 | 1 | 1 | 0 | 1 | 0 |
| R_gyrus_rectus_Volume | 1 | 1 | 0 | 2 | 1 | 1 |
| R_hippocampus_AvgMeanCurvature | 1 | 0 | 0 | 1 | 1 | 0 |
| R_hippocampus_ComputeArea | 1 | 1 | 0 | 2 | 0 | 2 |
| R_hippocampus_Curvedness | 0 | 1 | 0 | 1 | 1 | 0 |
| R_hippocampus_Volume | 0 | 1 | 0 | 1 | 0 | 1 |
| R_inferior_frontal_gyrus_ComputeArea | 1 | 0 | 0 | 1 | 0 | 1 |
| R_inferior_frontal_gyrus_Volume | 2 | 1 | 2 | 1 | 0 | 3 |
| R_inferior_occipital_gyrus_ComputeArea | 2 | 0 | 1 | 1 | 1 | 1 |
| R_inferior_temporal_gyrus_ComputeArea | 0 | 1 | 1 | 0 | 0 | 1 |
| R_inferior_temporal_gyrus_Volume | 0 | 1 | 1 | 0 | 0 | 1 |
| R_insular_cortex_Curvedness | 1 | 1 | 2 | 0 | 0 | 2 |
| R_insular_cortex_ShapeIndex | 0 | 1 | 1 | 0 | 0 | 1 |
| R_insular_cortex_Volume | 2 | 2 | 2 | 2 | 1 | 3 |
| R_lateral_orbitofrontal_gyrus AvgMeanCurvature | 0 | 1 | 1 | 0 | 1 | 0 |
| R_lateral_orbitofrontal_gyrus_ComputeArea | 1 | 0 | 1 | 0 | 0 | 1 |
| R_lateral_orbitofrontal_gyrus_ShapeIndex | 1 | 0 | 1 | 0 | 1 | 0 |
| R_lingual_gyrus_AvgMeanCurvature | 1 | 1 | 0 | 2 | 2 | 0 |
| R_lingual_gyrus_ComputeArea | 0 | 1 | 0 | 1 | 1 | 0 |
| R_lingual_gyrus_Curvedness | 0 | 1 | 0 | 1 | 1 | 0 |
| R_middle_frontal_gyrus_ComputeArea | 1 | 0 | 0 | 1 | 0 | 1 |
| R_middle_frontal_gyrus_Curvedness | 1 | 0 | 1 | 0 | 0 | 1 |
| R_middle_frontal_gyrus_ShapeIndex | 1 | 0 | 1 | 0 | 1 | 0 |
| R_middle_occipital_gyrus_AvgMeanCurvature | 2 | 1 | 1 | 2 | 3 | 0 |
| R_middle_occipital_gyrus_ComputeArea | 0 | 1 | 1 | 0 | 0 | 1 |
| R_middle_occipital_gyrus_Curvedness | 1 | 3 | 2 | 2 | 3 | 1 |
| R_middle_occipital_gyrus_Volume | 0 | 1 | 1 | 0 | 0 | 1 |
| R_middle_orbitofrontal_gyrus AvgMeanCurvature | 1 | 0 | 1 | 0 | 1 | 0 |
| R_middle_temporal_gyrus_ComputeArea | 1 | 0 | 0 | 1 | 0 | 1 |
| R_parahippocampal_gyrus_AvgMeanCurvature | 0 | 1 | 0 | 1 | 1 | 0 |
| R_parahippocampal_gyrus_ComputeArea | 0 | 1 | 0 | 1 | 0 | 1 |
| R_parahippocampal_gyrus_Volume | 1 | 0 | 1 | 0 | 1 | 0 |
| R_postcentral_gyrus_AvgMeanCurvature | 1 | 1 | 2 | 0 | 1 | 1 |
| R_postcentral_gyrus_ComputeArea | 1 | 0 | 1 | 0 | 0 | 1 |
| R_postcentral_gyrus_Curvedness | 1 | 0 | 0 | 1 | 1 | 0 |
| R_precentral_gyrus_AvgMeanCurvature | 0 | 1 | 1 | 0 | 0 | 1 |
| R_precentral_gyrus_ComputeArea | 1 | 0 | 0 | 1 | 0 | 1 |
| R_precentral_gyrus_ShapeIndex | 1 | 0 | 1 | 0 | 1 | 0 |
| R_precentral_gyrus_Volume | 0 | 1 | 1 | 0 | 0 | 1 |
| R_precuneus_Curvedness | 1 | 0 | 0 | 1 | 1 | 0 |
| R_precuneus_Volume | 1 | 1 | 0 | 2 | 0 | 2 |
| R_putamen_AvgMeanCurvature | 0 | 1 | 1 | 0 | 0 | 1 |
| R_putamen_ComputeArea | 1 | 0 | 0 | 1 | 0 | 1 |
| R_putamen_ShapeIndex | 1 | 0 | 0 | 1 | 1 | 0 |
| R_putamen_Volume | 1 | 0 | 0 | 1 | 0 | 1 |
| R_superior_frontal_gyrus_ComputeArea | 1 | 2 | 2 | 1 | 1 | 2 |
| R_superior_frontal_gyrus_Curvedness | 1 | 0 | 0 | 1 | 0 | 1 |
| R_superior_frontal_gyrus_ShapeIndex | 0 | 1 | 1 | 0 | 1 | 0 |
| R_superior_occipital_gyrus_Volume | 1 | 1 | 2 | 0 | 0 | 2 |
| R_superior_parietal_gyrus_ShapeIndex | 0 | 1 | 0 | 1 | 1 | 0 |
| R_superior_temporal_gyrus_Curvedness | 1 | 0 | 0 | 1 | 0 | 1 |
| R_superior_temporal_gyrus_ShapeIndex | 1 | 1 | 0 | 2 | 2 | 0 |
| R_supramarginal_gyrus_AvgMeanCurvature | 1 | 0 | 0 | 1 | 1 | 0 |
| R_supramarginal_gyrus_ShapeIndex | 0 | 1 | 1 | 0 | 1 | 0 |
| R_supramarginal_gyrus_Volume | 1 | 0 | 0 | 1 | 0 | 1 |
| UPDRS_Part_I_Summary_Score_Baseline | 1 | 1 | 2 | 0 | 2 | 0 |
| UPDRS_Part_II_Patient_Questionnaire Summary_Score_Baseline | 2 | 2 | 2 | 2 | 4 | 0 |
| UPDRS_Part_III_Summary_Score_Baseline | 1 | 2 | 2 | 1 | 3 | 0 |

### Table C

Model Reliability (Analysis of “Variance Importance”)

- 1. **Table C** is based on the variable importance scores of the classification (**Top 30 variables**)
  2. The yellow highlight rows are the variables that rank top 30 of all 4 studies (2x2 design)

(balanced/unbalanced vs. patient-group, PD vs. PD+SWEDD).

- 1. Correspondingly, blue indicates 3 times and green indicates 2 times.
  2. In summary, the same classification without UPDRS scores is also created. The graphs, table and spreadsheet for details

**Notes**:

- UPDRS measures (that are naturally longitudinal) are averaged in this analysis! Will explore alternatives (see change-based modeling in *further analyses* below).
- Only used imaging measures (for patients that did have repeated measures) at baseline. Will expand that (see further analyses below).

**Table C:**  Most reliable PD predictors for AbaBoost diagnostic classification.

| **Repeated times (2x2)**  **(balanced vs. patient-group)** | **Variable names (with UPDRS)** | **Variable names (without UPDRS)** |
| --- | --- | --- |
| **4** | **Number of variables=4**  L_hippocampus_ComputeArea  L_hippocampus_Volume  L_parahippocampal_gyrus_ComputeArea  UPDRS_Part_II_Patient_Questionnaire_Summary_Score_Baseline | **Number of variables=1**  L_inferior_frontal_gyrus_Volume |
| **3** | **Number of variables=8**  L_hippocampus_ShapeIndex  L_lateral_orbitofrontal_gyrus_Volume  L_parahippocampal_gyrus_Curvedness  L_parahippocampal_gyrus_Volume  L_superior_occipital_gyrus_ShapeIndex  R_middle_occipital_gyrus_AvgMeanCurvature  R_middle_occipital_gyrus_Curvedness  UPDRS_Part_III_Summary_Score_Baseline | **Number of variables =7**  L_insular_cortex_ComputeArea  L_middle_occipital_gyrus_Volume  L_parahippocampal_gyrus_Volume  L_putamen_ComputeArea  L_putamen_Volume  R_inferior_frontal_gyrus_Volume  R_insular_cortex_Volume |
| **2** | **Number of variables=15**  Age  cerebellum_AvgMeanCurvature  cerebellum_Curvedness  cerebellum_Volume  L_fusiform_gyrus_ShapeIndex  L_gyrus_rectus_AvgMeanCurvature  L_gyrus_rectus_ShapeIndex  L_hippocampus_Curvedness  L_inferior_temporal_gyrus_AvgMeanCurvature  L_inferior_temporal_gyrus_ShapeIndex  L_lateral_orbitofrontal_gyrus_ShapeIndex  L_precentral_gyrus_AvgMeanCurvature  R_lingual_gyrus_AvgMeanCurvature  R_superior_temporal_gyrus_ShapeIndex  UPDRS_Part_I_Summary_Score_Baseline | **Number of variables =17**  L_hippocampus_AvgMeanCurvature  L_hippocampus_ComputeArea  L_hippocampus_Volume  L_inferior_frontal_gyrus_ComputeArea  L_lateral_orbitofrontal_gyrus_ComputeArea  L_lingual_gyrus_AvgMeanCurvature  L_middle_frontal_gyrus_Curvedness  L_middle_occipital_gyrus_ComputeArea  L_middle_orbitofrontal_gyrus_ComputeArea  L_precuneus_Curvedness  L_putamen_AvgMeanCurvature  R_gyrus_rectus_ComputeArea  R_hippocampus_ComputeArea  R_insular_cortex_Curvedness  R_precuneus_Volume  R_superior_frontal_gyrus_ComputeArea  R_superior_occipital_gyrus_Volumebrainstem_ComputeArea |

### Table 5

Table 5: Between-cohort differences in some demographic, genetic and clinical variables.

| Variable | Categories/Classes | HC | PD | SWEDD | Exact Fisher's Test (p-value) |
| --- | --- | --- | --- | --- | --- |
| sex | 1 | 84 | 170 | 23 | 0.711 |
|  | 2 | 39 | 93 | 14 |  |
| chr12_rs34637584_GT | 0 | 123 | 259 | 37 | 0.523 |
|  | 1 | 0 | 4 | 0 |  |
| chr17_rs11868035_GT | 0 | 67 | 127 | 15 | 0.189 |
|  | 1 | 40 | 107 | 20 |  |
|  | 2 | 16 | 29 | 2 |  |
| chr17_rs11012_GT | 0 | 84 | 176 | 28 | 0.733 |
|  | 1 | 37 | 78 | 8 |  |
|  | 2 | 2 | 9 | 1 |  |
| chr17_rs393152_GT | 0 | 77 | 160 | 26 | 0.904 |
|  | 1 | 40 | 89 | 10 |  |
|  | 2 | 6 | 14 | 1 |  |
| chr17_rs12185268_GT | 0 | 80 | 163 | 26 | 0.906 |
|  | 1 | 39 | 88 | 10 |  |
|  | 2 | 4 | 12 | 1 |  |
| chr17_rs199533_GT | 0 | 82 | 167 | 26 | 0.779 |
|  | 1 | 39 | 86 | 10 |  |
|  | 2 | 2 | 10 | 1 |  |
| COGSTATE | Dementia (PDD) | 0 | 5 | 0 | **7.60E-08** |
|  | Mild Cognitive Impairment (PD-MCI) | 2 | 46 | 12 |  |
|  | Normal Cognition (PD-NC) | 121 | 212 | 25 |  |
| COGDECLN | No | 121 | 230 | 31 | **0.00025322** |
|  | Yes | 2 | 33 | 6 |  |
| FNCDTCOG | No | 121 | 251 | 33 | **0.04540301** |
|  | Yes | 2 | 12 | 4 |  |
| COGDXCL | 10% - 49% | 0 | 5 | 0 | **0.000547062** |
|  | 50% - 89% | 5 | 45 | 7 |  |
|  | 90% - 100% | 118 | 213 | 30 |  |

### Table 7

Table 7: GEE and GLMM predictive model summaries.

| GEE | GLMM |
| --- | --- |
| \|  \| **Estimate** \| **Std.err** \| **Wald** \| **Pr(>\|W\|)** \| \| --- \| --- \| --- \| --- \| --- \| \| (Intercept) \| 1.117257 \| 0.723184 \| 2.39 \| 0.12237 \| \| L superior parietal gyrus ComputeArea \| 1.400402 \| 1.497027 \| 0.88 \| 0.34955 \| \| L superior parietal gyrus Volume \| -2.525902 \| 1.359074 \| 3.45 \| 0.06309 \| \| R superior parietal gyrus ComputeArea \| 1.164381 \| 0.976521 \| 1.42 \| 0.23311 \| \| R superior parietal gyrus Volume \| -0.451386 \| 1.007911 \| 0.2 \| 0.65427 \| \| L putamen ComputeArea \| 0.496793 \| 0.713507 \| 0.48 \| 0.48626 \| \| L putamen Volume \| -0.61937 \| 0.83991 \| 0.54 \| 0.46086 \| \| R putamen Volume \| 0.836431 \| 0.946288 \| 0.78 \| 0.37675 \| \| R putamen ShapeIndex \| 0.56228 \| 0.354731 \| 2.51 \| 0.11295 \| \| L caudate ComputeArea \| -0.413876 \| 1.746183 \| 0.06 \| 0.81264 \| \| L caudate Volume \| 1.580367 \| 1.540474 \| 1.05 \| 0.30494 \| \| R caudate ComputeArea \| 0.155381 \| 1.410379 \| 0.01 \| 0.91227 \| \| R caudate Volume \| -1.804119 \| 1.502705 \| 1.44 \| 0.22991 \| \| **chr12 rs34637584 GT** \| **35.800527** \| **2.982947** \| **144.04** \| **<2.00E-16** \| \| chr17 rs11868035 GT \| -0.716443 \| 0.407688 \| 3.09 \| 0.07886 \| \| chr17 rs11012 GT \| -0.431071 \| 0.891313 \| 0.23 \| 0.62864 \| \| **chr17 rs393152 GT** \| **-1.230197** \| **0.594327** \| **4.28** \| **0.03846** \| \| **chr17 rs12185268 GT** \| **2.556489** \| **1.024266** \| **6.23** \| **0.01256** \| \| chr17 rs199533 GT \| -0.883482 \| 0.889914 \| 0.99 \| 0.32082 \| \| **Sex** \| **1.808176** \| **0.577873** \| **9.79** \| **0.00175** \| \| Weight \| 0.473417 \| 0.307536 \| 2.37 \| 0.12371 \| \| **Age** \| **-1.332754** \| **0.330977** \| **16.21** \| **5.70E-05** \| \| **UPDRS Part I Summary Score Baseline** \| **-3.380284** \| **0.72056** \| **22.01** \| **2.70E-06** \| \| **UPDRS Part II Patient Questionnaire Summary Score Baseline** \| **7.210265** \| **1.560543** \| **21.35** \| **3.80E-06** \| \| **UPDRS Part III Summary Score Baseline** \| **6.603029** \| **2.144869** \| **9.48** \| **0.00208** \| \| FID IID \| 0.000757 \| 0.001309 \| 0.33 \| 0.56317 \| \| **COGSTATE** \| **-7.918928** \| **1.672468** \| **22.42** \| **2.20E-06** \| \| **COGDECLN** \| **-4.720291** \| **1.398451** \| **11.39** \| **0.00074** \| \| **FNCDTCOG** \| **32.085919** \| **6.647111** \| **23.3** \| **1.40E-06** \| \| COGDXCL \| -0.489857 \| 1.434844 \| 0.12 \| 0.7328 \| \| EDUCYRS \| 0.07841 \| 0.099566 \| 0.62 \| 0.43098 \|   Estimated Correlation Parameters  Estimate Std.err  alpha 0.000159 0.000108  Number of clusters: 423 Maximum cluster size: 3 | **AIC      BIC   logLik deviance df.resid**     193.8    380.9  -64.9    129.8     2530  **Fixed Effects**:   \|  \| **Estimate** \| **Pr(>\|z\|)** \| \| --- \| --- \| --- \| \| (Intercept) \| -7.82E+01 \| 0.99904 \| \| L_superior_parietal_gyrus_ComputeArea \| -1.94E+00 \| 0.77698 \| \| L_superior_parietal_gyrus_Volume \| 2.34E+00 \| 0.74594 \| \| R_superior_parietal_gyrus_ComputeArea \| -1.36E-01 \| 0.98267 \| \| R_superior_parietal_gyrus_Volume \| 1.31E+00 \| 0.84431 \| \| L_putamen_ComputeArea \| -3.63E+00 \| 0.52797 \| \| L_putamen_Volume \| 2.84E+00 \| 0.58167 \| \| R_putamen_Volume \| -2.95E-01 \| 0.93113 \| \| R_putamen_ShapeIndex \| -2.06E-01 \| 0.87846 \| \| L_caudate_ComputeArea \| -6.77E+00 \| 0.39352 \| \| L_caudate_Volume \| 3.38E+00 \| 0.65557 \| \| R_caudate_ComputeArea \| 5.30E+00 \| 0.53451 \| \| R_caudate_Volume \| -2.08E+00 \| 0.78918 \| \| chr12_rs34637584_GT \| -4.51E+00 \| 0.99272 \| \| chr17_rs11868035_GT \| -4.92E-01 \| 0.76636 \| \| chr17_rs11012_GT \| 7.34E-01 \| 0.82913 \| \| chr17_rs393152_GT \| 2.92E+00 \| 0.39461 \| \| chr17_rs12185268_GT \| 3.02E+00 \| 0.60026 \| \| chr17_rs199533_GT \| -2.90E+00 \| 0.55589 \| \| Sex \| 1.09E+00 \| 0.68982 \| \| Weight \| 1.88E+00 \| 0.16203 \| \| Age \| 1.83E+00 \| 0.16687 \| \| UPDRS_part_I \| 3.29E-01 \| 0.84611 \| \| **UPDRS_part_II** \| **-1.28E+01** \| **0.0014** \| \| **UPDRS_part_III** \| **-2.09E+01** \| **0.00026** \| \| FID_IID \| -1.60E-03 \| 0.62892 \| \| COGSTATE \| 9.42E+00 \| 0.23984 \| \| COGDECLN \| -4.48E+00 \| 0.27759 \| \| FNCDTCOG \| 8.71E+00 \| 0.99989 \| \| COGDXCL \| -4.27E+00 \| 0.39832 \| \| EDUCYRS \| 3.68E-02 \| 0.97715 \| |

Legend: GEE= generalized estimating equation; GLMM=generalized linear mixed model; AIC= Akaike information criterion; BIC= Bayesian information criterion; logLik=log-likelihood function.

### Table 10

| Weight | Using UPDRS | Weight | Without UPRDS |
| --- | --- | --- | --- |
| 2.47 | **UPDRS_Part_III_Summary_Score_Baseline**  UPDRS_Part_III_Summary_Score_Baseline <= 8.94 : 1  UPDRS_Part_III_Summary_Score_Baseline > 8.94 : 0  UPDRS_Part_III_Summary_Score_Baseline is missing :0  **Class distributions**  UPDRS_Part_III_Summary_Score_Baseline <= 8.94  0 1  0.124 0.876  UPDRS_Part_III_Summary_Score_Baseline > 8.94  0 1  0.963 0.037  UPDRS_Part_III_Summary_Score_Baseline is missing  0 1  0.571 0.429 | **0.29** | **COGSTATE**  COGSTATE <= 2.5 : 0  COGSTATE > 2.5 : 0  COGSTATE is missing : 0  **Class distributions**  COGSTATE <= 2.5  0 1  0.976 0.0235  COGSTATE > 2.5  0 1  0.527 0.4729  COGSTATE is missing  0 1  0.571 0.428 |
| 1.9 | **UPDRS_Part_II_Patient_Q_Sum_Score_Base**  UPDRS_Part_II_Patient_Q_Sum_Score_Base <= 0.984 : 1  UPDRS_Part_II_Patient_Q_Sum_Score_Base > 0.984 : 0  UPDRS_Part_II_Patient_Q_Sum_Score_Base is missing :0  **Class distributions**  UPDRS_Part_II_Patient_Q_Sum_Score_Base <= 0.984  0 1  0.058 0.941  UPDRS_Part_II_Patient_Q_Sum_Score_Base > 0.984  0 1  0.846 0.154  UPDRS_Part_II_Patient_Q_Sum_Score_Base is missing  0 1  0.6515 0.349 | **0.43** | **L_insular_cortex_Curvedness**  L_insular_cortex_Curvedness <= 0.1053 : 0  L_insular_cortex_Curvedness > 0.1053 : 1  L_insular_cortex_Curvedness is missing : 0  **Class distributions**  L_insular_cortex_Curvedness <= 0.1053  0 1  0.786 0.214  L_insular_cortex_Curvedness > 0.1053  0 1  0.4744 0.526  L_insular_cortex_Curvedness is missing  0 1  0.5693 0.431 |
| 1.38 | **Age**  Age <= 72 : 0  Age > 72 : 1  Age is missing : 0  **Class distributions**  Age <= 72  0 1  0.76 0.236  Age > 72.0507  0 1  0.08 0.91  Age is missing  0 1  0.61 0.39 | **0.47** | **EDUCYRS**  EDUCYRS <= 12.06716133700685 : 0  EDUCYRS > 12.06716133700685 : 0  EDUCYRS is missing : 0  **Class distributions**  EDUCYRS <= 12.067  0 1  0.943 0.057  EDUCYRS > 12.067  0 1  0.571 0.429  EDUCYRS is missing  0 1  0.6153 0.385 |
| 1.0 | **cerebellum_ComputeArea**  cerebellum_ComputeArea <= 18739 : 1  cerebellum_ComputeArea > 18739 : 0  cerebellum_ComputeArea is missing : 1  **Class distributions**  cerebellum_ComputeArea <= 18739  0 1  0.287 0.7126  cerebellum_ComputeArea > 18739  0 1  0.815 0.1847  cerebellum_ComputeArea is missing  0 1  0.3854 0.6146 | **0.61** | **L_inferior_frontal_gyrus_AvgMeanCurvature**  L_inferior_frontal_gyrus_AvgMeanCurvature <= 0.089 : 1  L_inferior_frontal_gyrus_AvgMeanCurvature > 0.09 : 0  L_inferior_frontal_gyrus_AvgMeanCurvature is missing : 0  **Class distributions**  L_inferior_frontal_gyrus_AvgMeanCurvature <= 0.09  0 1  0.389 0.611  L_inferior_frontal_gyrus_AvgMeanCurvature > 0.09  0 1  0.668 0.332  L_inferior_frontal_gyrus_AvgMeanCurvature is missing  0 1  0.5678 0.4322 |
| Overall | Correctly Classified Instances 828 96.1672 %  Incorrectly Classified Instances 33 3.8328 %  Kappa statistic 0.9218  Mean absolute error 0.0556  Root mean squared error 0.1693  Relative absolute error 11.3517 %  Root relative squared error 34.2183 %  Total Number of Instances 861  TP Rate FP Rate Precision Recall F-Measure ROC Class  0.965 0.043 0.967 0.965 0.966 0.994 0  0.957 0.035 0.954 0.957 0.955 0.994 1  Confusion Matrix  a b <-- classified as  475 17 \| a = 0  16 353 \| b = 1 | **Overall** | Correctly Classified Instances 611 70.964 %  Incorrectly Classified Instances 250 29.036 %  Kappa statistic 0.4048  Mean absolute error 0.3838  Root mean squared error 0.4303  Relative absolute error 78.3447 %  Root relative squared error 86.96 %  Total Number of Instances 861  TP Rate FP Rate Precision Recall F-Measure ROC Class  0.758 0.355 0.74 0.758 0.749 0.781 0  0.645 0.242 0.667 0.645 0.656 0.781 1  Confusion Matrix  a b <-- classified as  373 119 \| a = 0  131 238 \| b = 1 |

### Figure 7

|  | with UPDRS data | without UPDRS data |
| --- | --- | --- |
| (default) imbalanced groups |  |  |
| rebalanced groups |  |  |
